# The key metabolic genes and networks regulating the fruit acidity and flavonoid of *Prunus mume* revealed via transcriptomic and metabolomic analyses

**DOI:** 10.3389/fpls.2025.1544500

**Published:** 2025-01-31

**Authors:** Xuan Gao, Shuangshuang Wu, Guosheng Lv, Mengyang Wang, Lingxiang Li, Yahui Liu, Feng He, Jiaxin Xiao

**Affiliations:** Anhui Provincial Key Laboratory of Biodiversity Conservation and Ecological Security in the Yangtze River Basin, College of Life Sciences, Anhui Normal University, Wuhu, China

**Keywords:** *Prunus mume*, fruit quality, citric acid, flavonoid, metabolic genes

## Abstract

The acidic taste of Mei fruit (*Prunus mume*) is a major contributor to its quality, but its formation mechanism remains unclear. Here, we unraveled the networks of organic acid and flavonoid metabolism in two Mei fruit. The results showed that the differentially expressed genes were mainly concentrated in the processes of carbohydrate derivative binding, carboxylic acid, and organic acid metabolism. While the differentially accumulated metabolites were mainly associated with flavone and flavonol biosynthesis and amino acid and carbon metabolism. Moreover, we identified key metabolites, such as citric and succinic acids, which may be central to the development of acidity in Mei fruit, and determined that they are under the regulatory influence of specific genes, including *galactinol-sucrose-galactosyltransferase 5*, *mitogen-activated protein kinase kinase kinase NPK1-like*, *glutamate receptor*, and *chalcone isomerase*. Furthermore, transcription factors *ERF027*, *bHLH92*, *bHLH35*, and *WRKY23* were identified as potential pivotal regulators within these networks. These results provide new insights into the metabolic regulation of acidity and flavonoid in Mei fruit.

## 1 Introduction

Mei (*Prunus mume* Siebold & Zucc.), also called green plum in China and Japanese apricot in Japan, originated in China and has been recorded in many ancient books, such as “The Book of Songs,” “Erya,” “Compendium of Materia Medica,” and “ShenNong’s Herbal Classic” (Chen, 2007). Mei is mainly distributed in China, South Korea, Japan, and other East Asian regions, and in Europe and the United States. Mei, cultivated in China for over 7,000 years, has rich germplasm resources, predominantly distributed in the Yangtze River basin and Southwestern and Southern China. A traditional fruit with both medicinal and food values, the Mei fruit is rich in dietary fiber, organic acids, amino acids, sugars, polyphenols, vitamins, and minerals (Liu et al., 2022). The citric and malic acid contents are relatively high in the fruit, and they are also relatively rich in essential amino acids and polyphenolic flavonoids. The fruit has anti-oxidative, antibacterial, anti-inflammatory (Pan et al., 2016; Pi and Lee, 2017), anti-viral (Ikeda et al., 2020; Nishide et al., 2019), anti-cancer (Nishi et al., 2020; Bailly, 2020; Zhao et al., 2023), and anti-obesity (Xia et al., 2010) properties, and blood pressure lowering and other health-related effects (Jo et al., 2023). Owing to its high nutritional and medicinal values, the fruit is known as “the king of cold fruit” and the “natural green health food” (Usenik et al., 2008).

Flavor is a critical determinant of food quality, significantly influencing consumer perception and preferences (Kim et al., 2023). The flavor of a food is an extremely complex characteristic, involving organic acids, sugars, amino acids, and numerous other secondary metabolites. For fruit, the flavor is greatly affected by the components of organic acids and soluble sugars (Xiong et al., 2020; Zhou et al., 2023). Organic acids and soluble sugars are not only nutrients but also key indicators of fruit taste (Si et al., 2022). With the improvement in people’s living standards, consumers are able to pay more attention to fruit flavor. However, the compositions of organic acids and soluble sugars differ greatly among fruits. Mei fruit is a key component of leisure food consumption and has a significant presence in terms of product variety and processing factories in Wuhu, Anhui Province in China. Mei fruit is known for its unique tartness, which is a fundamental part of its appeal. The sugar and acidity contents in Mei fruit during the maturity phase play pivotal roles in determining the quality levels of the processed products. However, balancing this tartness with the right amount of sweetness is critical. This balance is largely determined by the fruit’s sugar and acid contents (Devi Ramaiya et al., 2013). On the other hand, flavonoids such as chlorogenic acid and catechins are common polyphenolic substances in the fruit, which not only affect the astringency of the fruit but also make important contributions to the overall flavor of the fruit (Zhang and Chen, 2007). Therefore, research into the acid and flavonoid contents of Mei fruit can provide valuable insights into how to optimize their flavor, thereby enhancing their overall quality and consumer appeal. By focusing on these key components, researchers can help to ensure that Mei fruit continue to delight consumers with their distinctive and desirable flavor profile. However, there is a significant knowledge gap regarding the changes in the primary acid and flavonoid contents during the harvest period and the genes implicated in the regulation of these content changes.

Understanding the differential regulation of organic acids and flavonoids cannot be understated, especially when considering its impact on the appeal of Mei fruit. Representing a crucial facet of fruit industry and consumption, the chemistry behind these fruit acids directly contributes to their unique flavors, aromas, and overall sensory profiles that captivate consumers’ palates globally. Consequently, in the context of Mei fruit, a revered fruit and botanical icon dating back thousands of years to ancient China, understanding the role and impact of organic acids and flavonoids becomes paramount. The balance of these components, specifically the relatively high citric and malic acid contents, and polyphenolic flavonoids in Mei fruit, significantly influences the fruit’s acidity, thereby determining its tartness. These flavor profiles are critical, directly feeding into the desirability of the fruit, shaping consumer preferences, and market demand. Hence, it is important to understand the differential regulation of organic acids and flavonoids within Mei fruit.

The integration of transcriptomic and metabolomic analyses presents numerous advantages in researching the formation and mechanisms of fruit flavor. Several studies have integrated metabolomic and transcriptomic analyses to investigate the mechanisms of flavor formation in fruit, including pineapple (*Ananas comosus*) (Chen et al., 2023; Gao et al., 2022), mango (*Mangifera indica*) (Datir and Regan, 2023), Satsuma mandarin (*Citrus unshiu*) (Duan et al., 2023), apricot (*Prunus armeniaca*) (Gou et al., 2023), persimmon (*Diospyros kaki*) (Han et al., 2023), oriental melon (*Cucumis melo*) (Cheng et al., 2022), and strawberry (*Fragaria ananassa*) (Liu et al., 2023). Key genes, metabolites, and metabolic pathways that contribute to the specific flavor profiles of these fruit have been identified. Metabolic pathways, such as sugar and acid metabolism, flavonoid and anthocyanin biosynthesis, carotenoid metabolism, and phenylpropanoid biosynthesis play critical roles in flavor formation. Key genes involved in these pathways have also been identified. Nevertheless, the metabolic genes and networks related to the acidity and flavonoid of Mei fruit have rarely been reported. Mei fruit attracts consumers with its unique acidic taste; however, the formation mechanism of this sour taste and its unique flavor is not clear. In this study, the fruit quality of the two different skin-colored Mei cultivars was evaluated, and the differences in genes and metabolites were analyzed based on transcriptomic and metabolomic data obtained from the fruit enlargement stage and green mature stage (i.e., harvesting period for processing). This study will provide a new perspective for understanding the flavor formation and offer a scientific basis for further regulation mechanism investigation related to flavor in Mei fruit.

## 2 Materials and methods

### 2.1 Plant Materials

Six-year-old “Changnong 17” (CN) and “Nanko” (NK) (*P. mume*) plants were collected from the Qiaocun Orchard (30°87′ N, 118°18′ E), Gongshan Town, Nanling County, Anhui Province. CN and NK are the two main cultivars of locally grown Mei, with similar flowering and maturity periods. CN is a domestic cultivar with a green skin, while NK is a cultivar introduced from Japan with a green skin and a faint blush. All the trees were spaced 3.4 m apart, and the rows were 3.2 m wide. In the same planting area, nine plants with relatively consistent height and crown width were randomly selected per cultivar; three plants formed a biological replicate, with three biological replicates. The CN and NK fruits were collected at the enlargement stage [23 April 2023, 62 days after full bloom (DAFB)], named CN1 and NK1, respectively, and green mature stage (7 May 2023, 76 DAFB), named CN3 and NK3, respectively (**Supplementary Figure S1**). At each sampling time, approximately five fruits of almost the same size were randomly selected from each tree, and approximately 15 fruits were collected per replicate, for a total of approximately 45 fruit collected per sample. Parts of each picked fruit were quickly frozen in liquid nitrogen. They were taken to the laboratory and stored at −80°C for the analyses of transcriptomes, metabolomes, genes [using quantitative real-time PCR (qPCR)], organic acids, sugars, free amino acids, and flavonoids. Another portion of each sample was taken to the laboratory in an ice box to determine the physiological indicators. The flowchart of the major analyses is shown in **Supplementary Figure S2**.

### 2.2 Fruit quality traits

A total of 10 fruits were randomly selected from each biological replicate to weigh using electron balance, and the mean value was calculated as the single fresh weight per biological replicate. The mass fraction of total soluble solids (TSS) was determined using a handheld sugar meter [portable refractometer (PAL-1)]. Titratable acidity (TA) content was determined by potentiometric titration (Yan and Jiang, 2012). Solid–acid ratio is equal to TSS divided by TA. Vitamin C (Vc) content was determined by colorimetric method (Zheng, 2006), and anthocyanin content was determined by pH differential method (Feng, 2016). The edible rate was calculated according to the proportion of pulp weight to the whole fruit weight. The longitudinal and transverse diameters of the fruit were measured with an Vernier caliper with an accuracy of 0.02 mm.

### 2.3 Transcriptome sequencing and data analysis

All samples were consigned to BGI TechSolutions Co., Ltd. for RNA extraction, library preparation, and sequencing using high-throughput sequencing technologies. RNA was extracted using the TRIzol reagent following the manufacturer’s protocol. After RNA quantification and quality assessment with a Bioanalyzer, library preparation was performed using the Illumina TruSeq RNA Sample Preparation Kit. Sequencing was conducted on an Illumina platform, generating paired-end reads. Raw reads were filtered using SOAPnuke for quality control to obtain clean reads. The clean reads were mapped to the genome of *Prunus mume* V1.0 using HISAT2 (Kim et al., 2015). The principal component analysis (PCA) was conducted using the R software package. By plotting the samples on a scatter plot based on their gene expression profiles, we verified the suitability of our samples for further analysis. We utilized the DESeq package in the R programming language to conduct an analysis of differential gene expression. The selection criteria for identifying differentially expressed genes (DEGs) were set at |log_2_Fold Change|>1 and FDR<0.05. DESeq is a widely recognized and extensively employed tool for detecting genes that exhibit differential expression patterns across different conditions. To gain insights into the biological functions and pathways associated with these DEGs, we performed gene annotation and enrichment analysis. The annotated genes were further subjected to gene ontology (GO) analysis, which categorizes genes into different functional groups based on their biological processes, cellular components, and molecular functions. Additionally, we carried out Kyoto Encyclopedia of Genes and Genomes (KEGG) analysis to identify the enriched pathways associated with the DEGs.

### 2.4 Metabolite profiling using UPLC-MS and data analysis

Briefly, 50 μg of samples was weighted into 1.5-mL tubes, followed by the addition of 1 mL of extraction solvent (methanol:water, 80:20, v/v). The mixture was vortexed, sonicated, and centrifuged to collect the supernatant. The supernatants were then subjected to ultra-performance liquid chromatography coupled with mass spectrometry (UPLC-MS). The metabolites were analyzed with waters 2777c UPLC (Waters, USA) in series with a Q exactive HF high-resolution mass spectrometer (Thermo Fisher Scientific, USA). For data processing, raw mass spectrometry data were processed using Compound Discoverer software (Thermo Fisher Scientific), which includes peak alignment, peak picking, and normalization. The variable importance for the project of orthogonal partial least squares discriminant analysis model ≥ 1, Fold Change ≤ 0.83 or ≥ 1.2, and *p*-value < 0.05 indicated differentially accumulated metabolites (DAMs). PCA analysis, volcano plot analysis, and heatmap analysis were performed to investigate the reliability of our sample data. Each sample was replicated six times. Our main objective was to explore the metabolic pathways enriched with differential metabolites in enlargement and green mature stages of the two Mei fruits, with a particular focus on sugar–acid metabolism and hormone metabolism. By employing KEGG analysis, we aimed to uncover the significant changes occurring in secondary metabolites during the ripening process of Mei fruits.

### 2.5 Weighted gene co-expression network analysis

For weighted gene co-expression network analysis (WGCNA), we utilized the WGCNA R package. The gene expression data were first normalized and log-transformed [log_2_(FPKM + 1)] to meet the assumptions of the analysis. Next, a suitable soft-thresholding power (β) was determined by constructing a scale-free topology model using the “pickSoftThreshold()” function, which calculates the appropriate power to achieve a scale-free network. The WGCNA dendrogram represents the clustering of genes into modules based on their expression patterns across samples. Each branch represents a gene, and the color bands indicate module assignments. Genes within the same module are presumed to be co-expressed and potentially co-regulated, suggesting that they may be involved in similar biological processes or pathways. Identifying these modules allows for the exploration of gene networks related to specific traits or conditions, such as Mei fruit ripening stages. The experiment incorporated samples from the two cultivars at two developmental stages, with three replicates per sample, resulting in 12 samples. These samples underwent WGCNA for both transcriptomic and metabolomic data. In the exploration of the regulatory networks influencing sugar–acid metabolism during Mei fruit maturation, differential genes were integrated from the maturity stages of the two fruiting Mei cultivars with genes from sugar–acid-related modules identified through the WGCNA. The shared genes and metabolites were further analyzed for their co-expression patterns using Spearman’s correlations with a threshold of 0.7 to ensure robust associations. Our network analysis, visualized using the Gephi software, revealed a complex interaction network.

### 2.6 Analyses of organic acids

Briefly, 1.0 g of frozen Mei fruit (CN3 and NK3) was ground into powder and then resuspended in 4 mL 0.1% phosphoric acid buffer solution. The mixtures were treated with an ice water ultrasonic bath for 30 min and centrifuged at 12,000 *g* for 5 min at 4°C. Each supernatant was re-extracted and combined twice. The final volume was set at 50 mL. Samples were filtered through 0.22-μm aqueous phase membrane. Organic acids were analyzed with Waters e2695 liquid chromatograph (Waters Corp., Milford, NH, USA) and a 2998 photoelectric secondary array detector equipped with a Polaris 180A C18-a chromatographic column (5 μm, 250 × 4.6 mm). The mobile phase was 0.5% NH_4_H_2_PO_4_ aqueous solution (pH=2.8). The flowrate was 1.0 mL/min, the injection volume was 10 μL, and the detection wavelength was 210 nm. The concentrations of fumaric acid and oxalic acid mixed standard solutions were 0.5 μg/mL, 1 μg/mL, 1.25 μg/mL, 2.5 μg/mL, 5 μg/mL, 10 μg/mL, 12.5 μg/mL, and 25 μg/mL, and the concentrations of malic acid, citric acid, and quinic acid mixed standard solutions were 10 μg/mL, 20 μg/mL, 25 μg/mL, 50 μg/mL, 100 μg/mL, 200 μg/mL, 250 μg/mL, and 500 μg/mL.

### 2.7 Analyses of sugars

To determine sugar content, 0.50 g of frozen CN3 and NK3 was ground into powder and then resuspended in 4 mL of 80% acetonitrile–water for full shaking. The mixtures were treated with a low-temperature water ultrasonic bath for 30 min and centrifuged at 12,000 *g* for 5 min at 4°C. Each supernatant was extracted again, and the supernatant was combined twice and diluted to 10 mL with 80% acetonitrile–water. Samples were filtered through 0.22 μM aqueous phase membrane. Sugars were analyzed with Agilent 1260A high-performance liquid chromatograph system (Agilent, Santa Clara, CA, USA) and ELSD evaporative light scattering detector equipped with a Poroshell 120 HILIC-Z chromatographic column (2.7 μm, 3 × 150 mm). The column temperature was 35°C. The mobile phase consisted of phase A (0.3% ammonia–water) and phase B (0.3% ammonia–acetonitrile). The gradient program was as follows: 0–8 min, 10% A, 90% B; 8–15 min, 10%–30% A, 90%–70% B; 15–19 min, 30% A, 70% B; 19–21 min, 30%–10% A, 70%–90% B; and 21–25 min, 10% A, 90% B. The flowrate was 0.6 mL/min, and the injection volume was 2 μL. The concentrations of sucrose, glucose, and fructose mixed standard solutions were 10 μg/mL, 20 μg/mL, 50 μg/mL, 100 μg/mL, 200 μg/mL, 500 μg/mL, and 1,000 μg/mL.

### 2.8 Analyses of amino acids

To analyze amino acid composition, 0.50 g of frozen CN3 and NK3 was ground into powder and then resuspended in 4 mL 20% ethanol including 0.001 M HCl, treated with a low-temperature water ultrasonic bath for 30 min, and centrifuged at 12,000 *g* for 5 min at 4°C. Each precipitate was extracted again, and the supernatant was combined twice and dilute to 10 mL. After fully mixing, samples were filtered through 0.22-μM aqueous phase membrane. Free amino acids were analyzed with Agilent 1260 liquid chromatograph tandem Xevo TQ’s mass spectrometer (Agilent, Santa Clara, CA, USA) equipped with a HILICZ chromatographic column (2.7 μm,3.0 × 100 mm). The column temperature was 35°C. The mobile phase consisted of two solvents: solvent A (75% acetonitrile-water) and solvent B (0.1 mol/L sodium acetate). The gradient program was as follows: 0–1 min, 10% A, 90% B; 1–5 min, 10%–50% A, 90%–50% B; 5–8 min, 50% A, 50% B; 8–8.1 min, 50%–10% A, 50%–90% B; and 8.1–15 min, 10% A, 90% B. The flowrate was 0.3 mL/min, and the injection volume was 1 μL. The concentrations of four amino acids (tryptophan, asparagine, glutamine, and cysteine) standard solutions were 10 ng/mL, 25 ng/mL, 50 ng/mL, 250 ng/mL, 500 ng/mL, 500 ng/mL, and 5,000 ng/mL, and the concentrations of other 16 amino acids standard solutions were 0.1 nmol/mL, 0.25 nmol/mL, 0.5 nmol/mL, 2.5 nmol/mL, 5 nmol/mL, 25 nmol/mL, and 50 nmol/mL.

### 2.9 Analyses of flavonoids

Briefly, 1.0 g of frozen CN3 and NK3 was ground into powder and then resuspended in 4 mL 1% HCl–methanol solution. Samples were then vortexed for 1 min, treated with an ice water ultrasonic bath for 30 min, and centrifuged at 11,000 *g* for 30 min. Each precipitate was extracted again, and the supernatant was combined twice. The final volume was set at 10 mL. After fully mixing, samples were filtered through a 0.22-μM organic phase membrane. Flavonoids were determined using an Agilent 1260 high-performance liquid chromatograph system in tandem with a Xevo TQ’s mass spectrometer (Agilent, Santa Clara, CA, USA) equipped with an Agilent ZORBAX Eclipse Plus C18 chromatographic column (3.5 µm, 2.1 × 150 mm). The column temperature was 35°C, automatic sampler temperature was 4°C, and operation time was 10 min. The mobile phase consisted of two solvents: solvent A, H_2_O (0.3% formic acid) and solvent B, acetonitrile (0.3% formic acid). The gradient program was as follows: 0–1 min, 80%–10% A, 20%–90% B; 1–5 min, 10% A and 90% B; 5–5.1 min, 10%–80% A, 90%–20% B; and 5.1–10 min, 80% A and 20% B. The flowrate was 0.3 mL/min, and the injection volume was 3 μL. The standard solution concentrations of flavonoids were 0.5 ng/mL, 2 ng/mL, 5 ng/mL, 20 ng/mL, 50 ng/mL, 200 ng/mL, 500 ng/mL, and 2,000 ng/mL.

### 2.10 qPCR validation

A total of eight genes related to organic acid and flavonoid metabolisms were selected for analysis using qPCR. All primers are listed in **Supplementary Table S1**. The expression levels of mRNAs were normalized to *LOC103320135*, which was selected based on its high and stable expression across all samples as determined from transcriptome sequencing data. This gene showed minimal variance under the experimental conditions, making it suitable as a reference gene for normalization. The relative gene expression levels were calculated using the 2^−ΔΔCT^ method (Livak and Schmittgen, 2001). The qPCR analysis of each sample was performed in triplicate.

### 2.11 Statistical analyses

Using IBM SPSS Statistics 26 and Miscrosoft Excel 2016, Tukey multiple comparison tests and one-way ANOVA were carried out at the significance level of *p* < 0.05. The data of flavonoids and amino acids were analyzed using the two independent-sample t-test; values for *p* < 0.05 were considered significant difference. Pearson’s correlation analysis was performed to compare the qPCR expression profiles and FPKM values from the RNA-seq.

## 3 Results

### 3.1 Differences in Mei fruit quality traits

**Figure 1:**
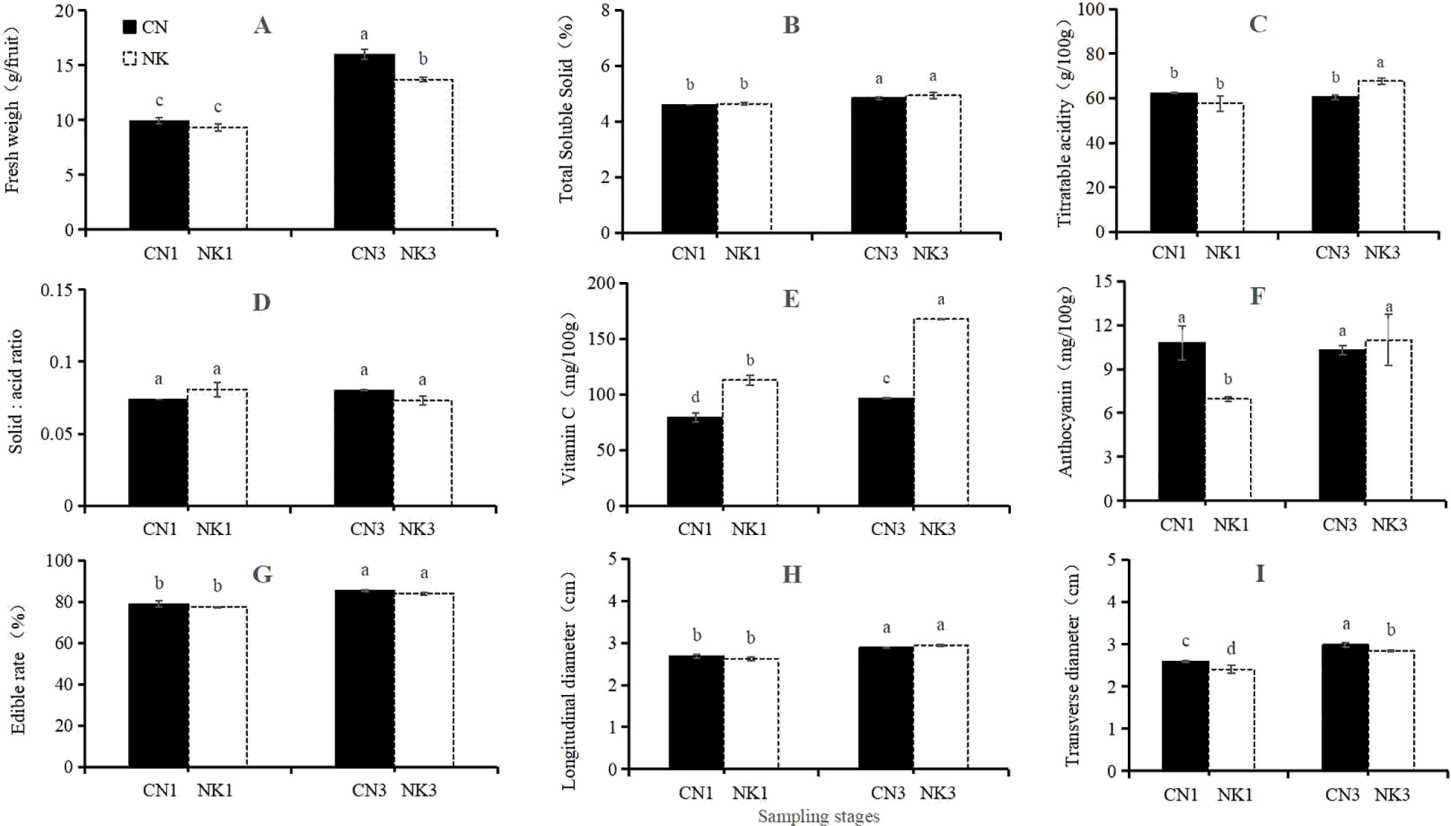
Fruit quality traits of ‘Changlong 17’ (CN) and ‘Nankao’ (NK) fruit at the enlargement stage and green mature stage. In panels **(A–I)**, fresh weigh, total soluble solids (TSS), titratable acidity (TA), solid–acid ratio, vitamin C (Vc), anthocyanin, edible rate, and longitudinal and transverse diameters are represented. Different lowercase letters indicate significant differences with *p*<0.05, as determined by Tukey’s test.

As shown in **Figure 1**, at 62 DAFB, the anthocyanin contents and transverse diameters of CN were significantly higher than those of NK, whereas the Vc content of CN was significantly lower than that of NK. There were no significant differences in the fresh weights, TSS and TA contents, solid–acid ratios, edible rates, and longitudinal diameters between the two cultivars. At 76 DAFB, the fresh weight and transverse diameter of CN were significantly higher than those of NK, whereas the TA and Vc contents of CN were significantly lower than those of NK. There were no significant differences in the TSS contents, solid–acid ratios, anthocyanin contents, edible rates, and longitudinal diameters between the two cultivars. From 62 to 76 DAFB, significant increases were observed for the fresh weights, transverse and longitudinal diameters, and edible rates of both Mei fruit, and the TA and anthocyanin contents of the NK. However, the Vc contents of both Mei fruit significantly declined, and no significant differences were found for the TSS contents and solid–acid ratios of both Mei fruit nor for the TA and anthocyanin contents of the CN.

### 3.2 DEGs across ripening stages

**Figure 2:**
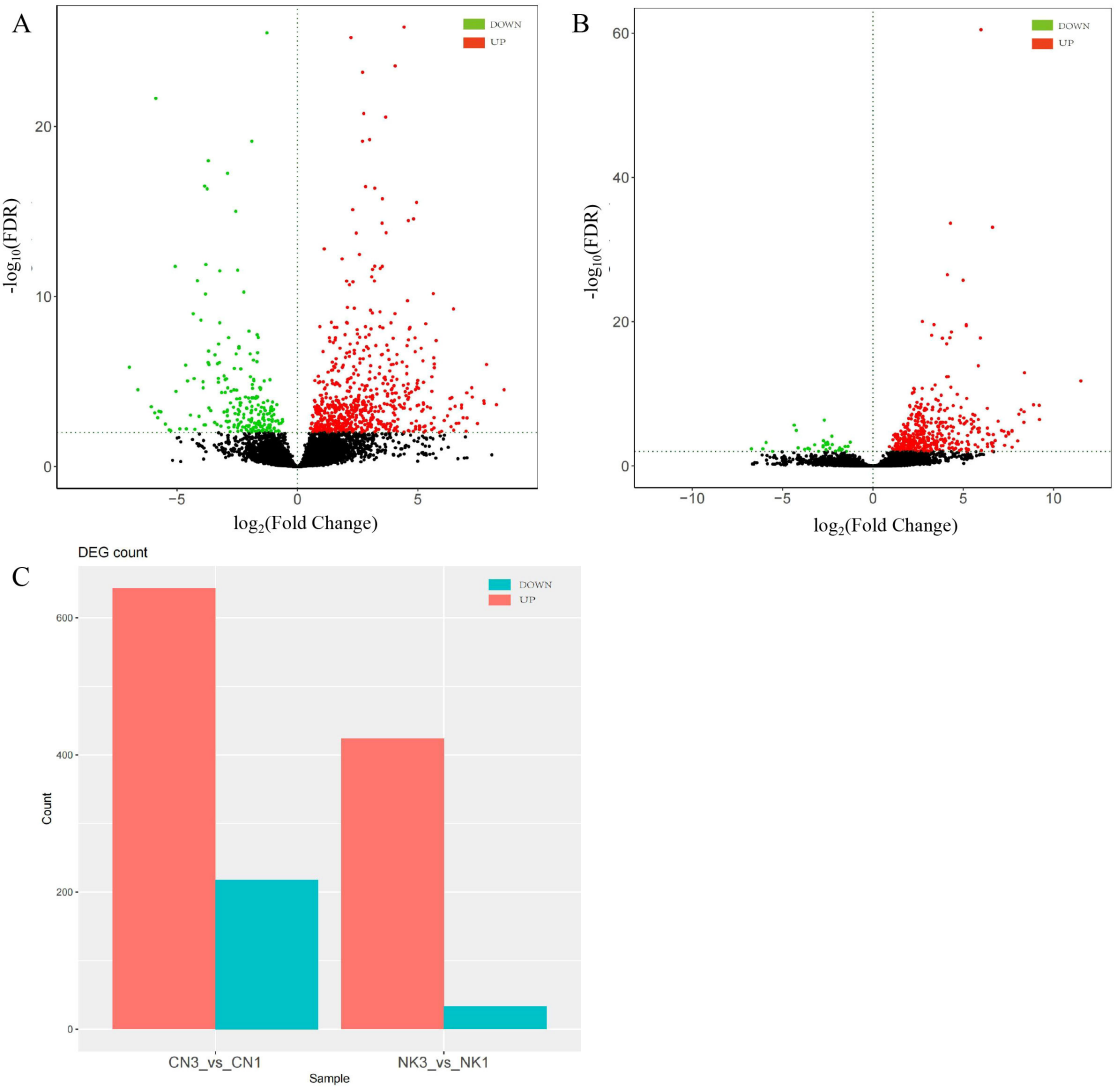
DEGs in ‘Changlong 17’ (CN) and ‘Nankao’ (NK) fruit across ripening stages. **(A)** Volcano plot of CN; **(B)** volcano plot of NK; **(C)** overview of expression levels of DEGs.

A total of 76.92 Gb of clean data was obtained. The Q30 base was distributed between 90.34% and 93.56%. By comparing to the reads of the reference genome, the genome alignment rate was 96.30%–96.85% (**Supplementary Table S2**). In our analysis of DEGs and metabolite profiling across ripening stages, we focused on genes related to flavor changes without prioritization based on expression fold changes. Employing stringent criteria of |log_2_Fold Change| > 1 and FDR < 0.01, we identified significant differences in gene expression between the enlargement and green mature stages for both CN and NK Mei fruit. For the CN cultivar, 861 DEGs were identified, with 643 genes upregulated and 218 genes downregulated as the fruit ripened. In contrast, the NK cultivar exhibited 457 DEGs, with 424 genes upregulated and 33 genes downregulated during ripening (**Figure 2**). Thus, the majority of genes were upregulated during the ripening process, with CN showing a greater number of upregulated genes compared with NK. This observation suggests that the ripening process in CN may involve a more extensive activation of certain genes related to flavor development, ripening, and possibly stress responses, compared with NK.

### 3.3 Transcriptomic profiling and PCA

**Figure 3:**
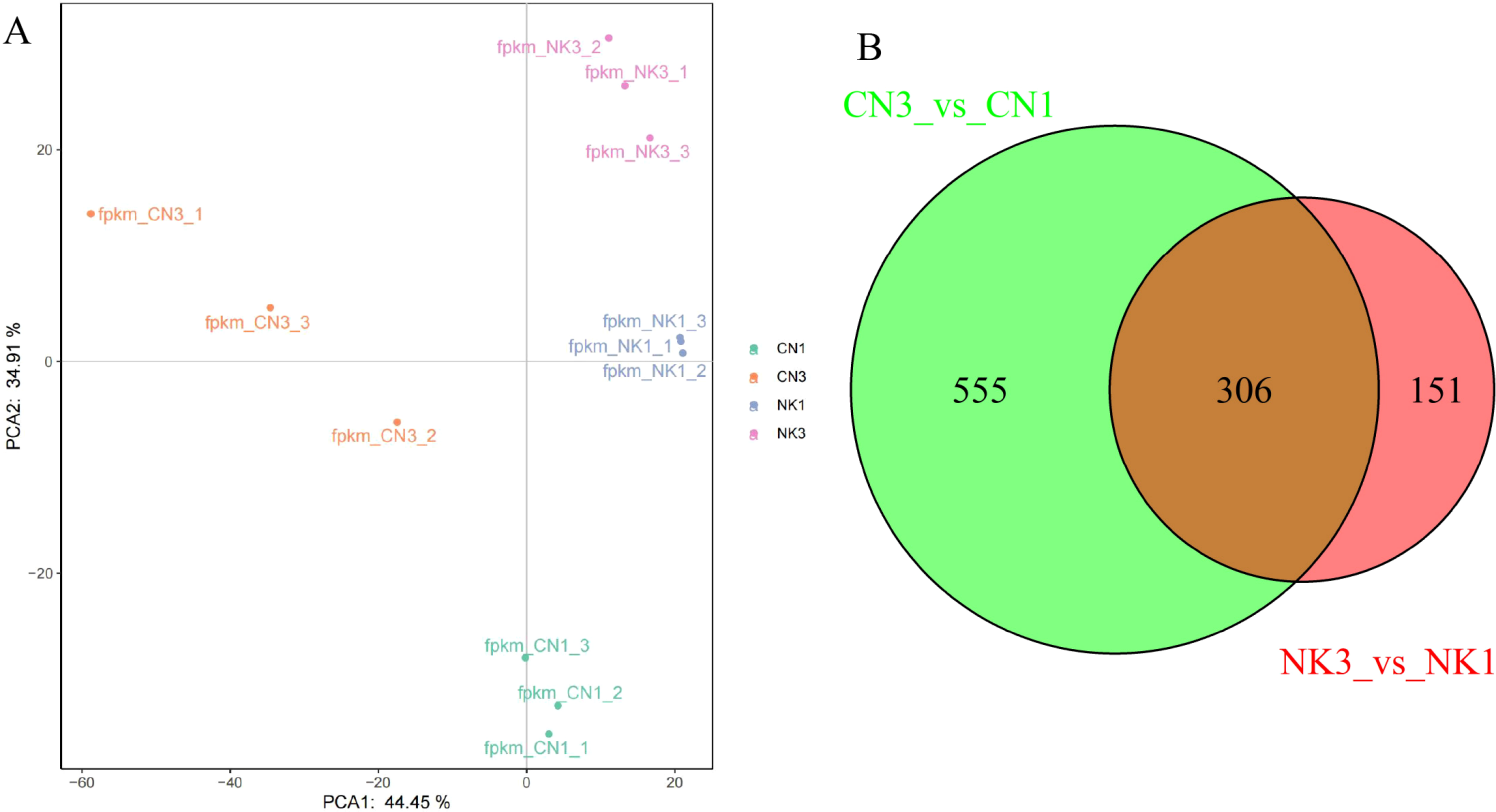
Transcriptomic analysis from ‘Changlong 17’ (CN) and ‘Nankao’ (NK) fruit across ripening stages. **(A)** PCA analysis of transcriptomic data; **(B)** Venn diagram illustrating the overlap of DEGs between CN and NK Mei fruit at development and green mature stages.

Based on the PCA derived from transcriptomic sequencing data, we observed a significant distinction between the CN and NK Mei fruit and their ripening stages. The PCA plot demonstrated that the first principal component (PCA1) and the second principal component (PCA2) accounted for 44.45% and 34.91% of the variance, respectively. Samples were well-clustered according to cultivar and ripening stage, which separated them into four distinct groups: CN1, CN3, NK1, and NK3 (**Figure 3A**). This clustering underscored the pronounced differences between the cultivars and suggested that varietal differences were more pronounced than those attributed to the ripening stage. There were no outliers, indicating consistent sample groupings and reinforcing the reliability of the transcriptomic data in reflecting the developmental changes and varietal distinctions among Mei fruit.

The transcriptomic profiling of CN and NK Mei fruit during ripening stages revealed distinctive and shared molecular signatures. CN fruit exhibited 555 unique DEGs, whereas NK fruit exhibited 151 unique DEGs. Importantly, 306 genes were shared between the two cultivars, suggesting common mechanisms or pathways critical for fruit ripening, such as flavor changes. Future analyses will focus on these shared DEGs to uncover processes central to ripening in both cultivars (**Figure 3B**).

### 3.4 GO Annotation and KEGG enrichment of common DEGs

**Figure 4:**
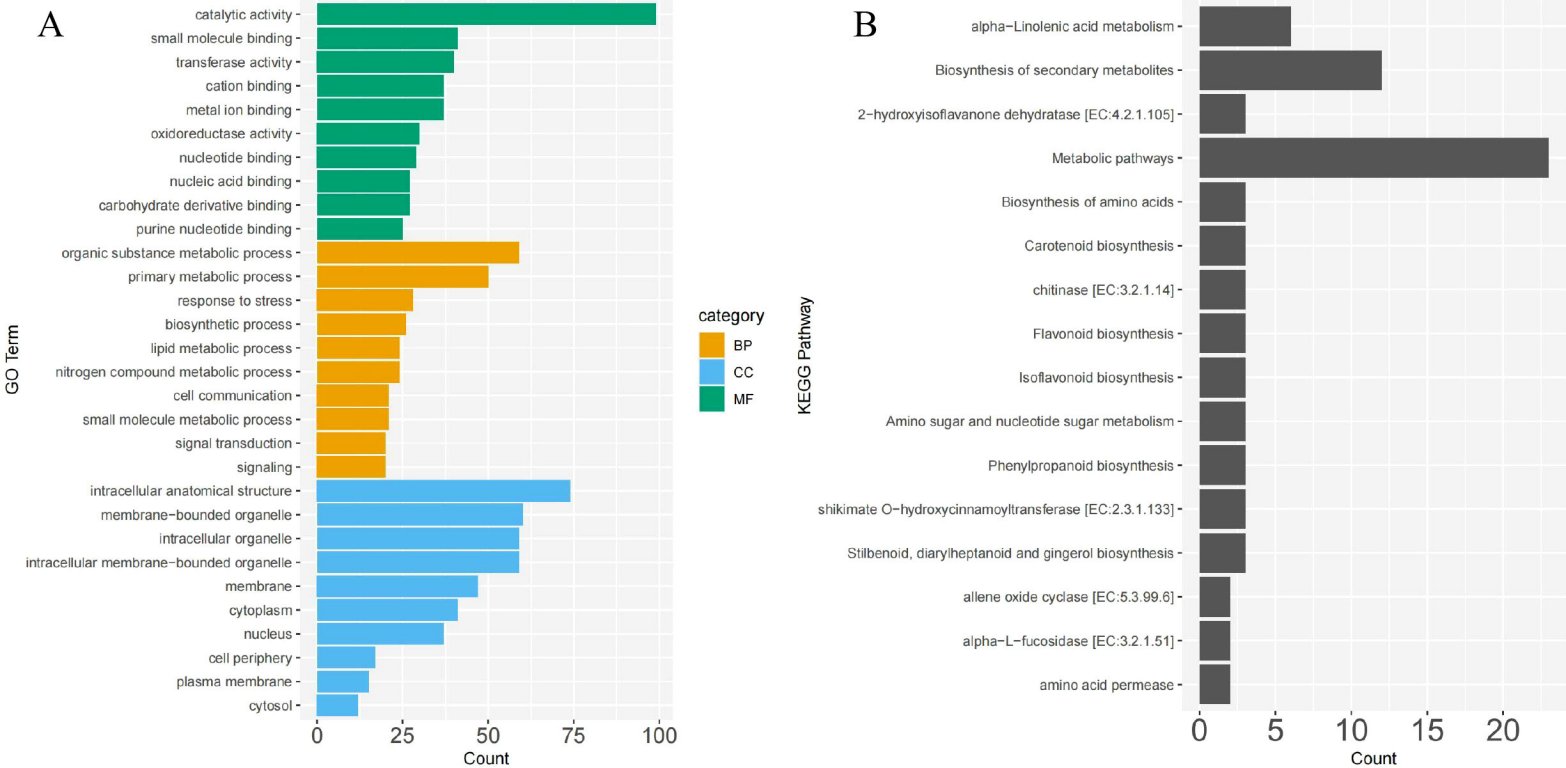
GO **(A)** and KEGG **(B)** pathway enrichment analysis of common DEGs between ‘Changlong 17’ (CN) and ‘Nankao’ (NK) fruit. BP, CC, and MF represent biological process, cellular component, and molecular function, respectively.

The GO annotation of DEGs shared between CN and NK Mei fruit revealed significant enrichments in several key functional categories relevant to the ripening process and flavor development. In the biological process category, genes were notably enriched in organic substance metabolic processes (59 genes), primary metabolic processes (50 genes), and response to stress (28 genes). Cellular component enrichments were primarily in intracellular anatomical structures (74 genes), membrane-bounded organelles (60 genes), and intracellular organelles (59 genes). For molecular functions, catalytic activity (99 genes), small molecule binding (41 genes), and transferase activity (40 genes) were predominant (**Figure 4A**). The molecular function category, particularly transferase activity, nucleic acid binding, and carbohydrate derivative binding, potentially relates to the metabolism of acids and sugars. Similarly, the organic substance metabolic process and primary metabolic process in the biological process category may be involved in the metabolism of volatile compounds related to aroma.

The KEGG pathway analysis of the DEGs shared between the CN and NK Mei fruit highlighted several key pathways involved in the ripening process and flavor development. Notable pathways included alpha-linolenic acid metabolism, biosynthesis of secondary metabolites, metabolic pathways, biosynthesis of amino acids, carotenoid biosynthesis, flavonoid biosynthesis, isoflavonoid biosynthesis, amino sugar and nucleotide sugar metabolism, phenylpropanoid biosynthesis, and stilbenoid, diarylheptanoid, and gingerol biosynthesis (**Figure 4B**). The enrichment of these pathways suggests a complex interplay of biochemical processes contributing to the unique flavor profiles of Mei fruit during ripening.

### 3.5 Metabolomic analysis of different Mei fruit developmental stages

**Figure 5:**
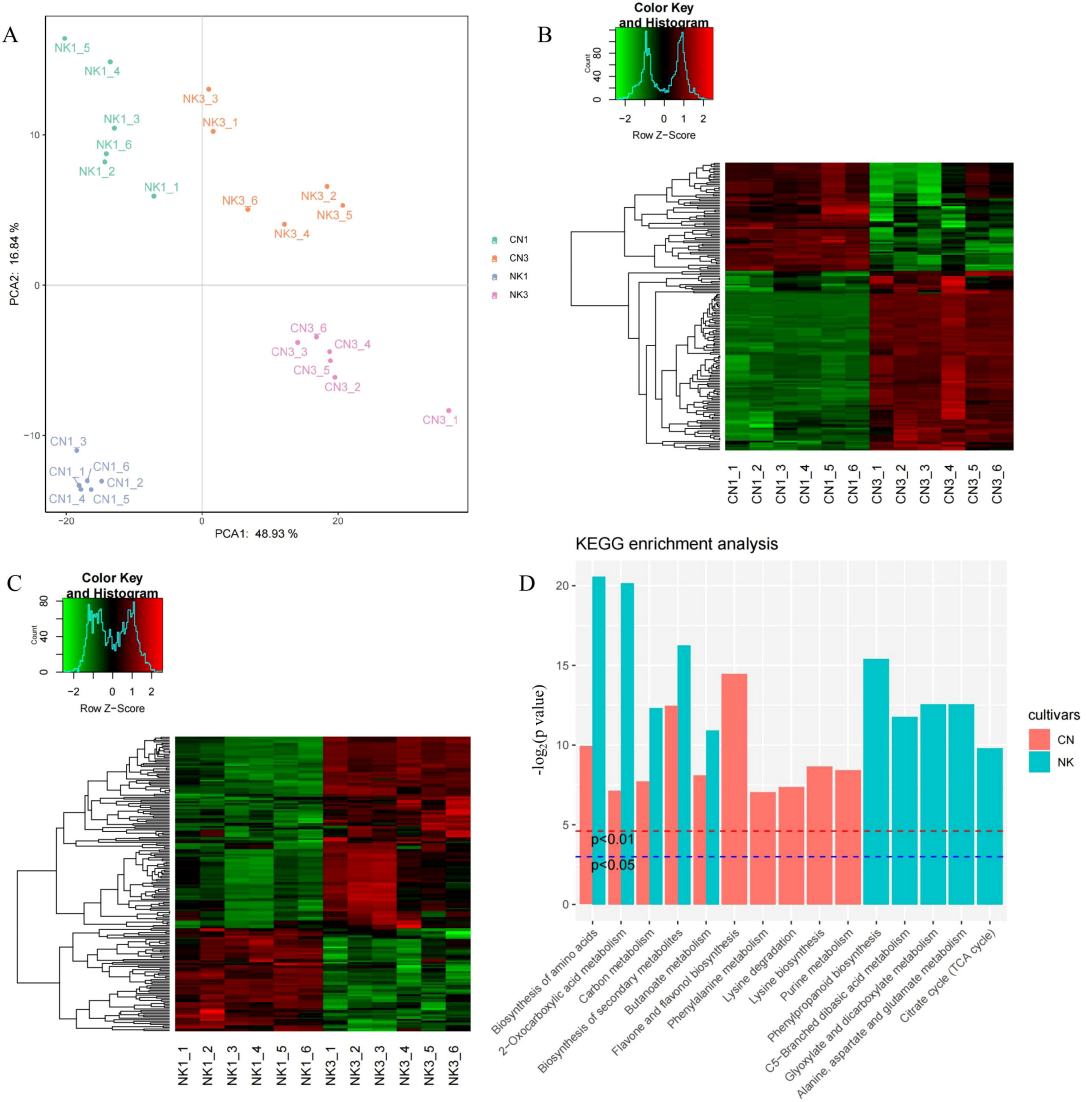
The analysis of metabolomic data for ‘Changlong 17’ (CN) and ‘Nankao’ (NK) fruit during ripening. **(A)** PCA analysis of all samples; **(B)** heatmaps of DAMs for CN; **(C)** heatmaps of DAMs for NK; **(D)** KEGG analysis of DAMs.

The PCA based on metabolomic data clearly segregated the CN and NK Mei fruit into four distinct groups corresponding to their variety and ripening stages (CN1, CN3, NK1, and NK3). The first principal component (PCA1) explained 48.93% of the variance, indicating a strong differentiation factor likely associated with the ripening stage, whereas the second principal component (PCA2) accounted for 16.84% of the variance, potentially reflecting variety-specific metabolic differences. This segregation underscored the profound impacts of ripening and varietal genetics on the metabolomic profile (**Figure 5A**). The metabolomic heatmaps for CN and NK across ripening stages revealed distinct expression patterns of metabolites associated with the maturation process. In both CN and NK cultivars, there were clear differentiations between enlargement and green mature stages, indicating significant changes in metabolite profiles as the fruit matures. This variation reflected the dynamic changes in primary and secondary metabolism, which potentially contribute to flavor development and other ripening-related physiological processes (**Figures 5B, C**). Both CN and NK Mei fruit share essential pathways involved in fruit growth-related metabolic processes, including biosynthesis of amino acids, 2-oxocarboxylic acid metabolism, carbon metabolism, biosynthesis of secondary metabolites, and butanoate metabolism (**Figure 5D**). These shared pathways underline common biochemical processes fundamental to ripening. Pathways like flavone and flavonol biosynthesis and purine metabolism were unique to CN, suggesting that these specific metabolic routes influenced the flavor profile of CN. NK exhibited exclusive enrichment in phenylpropanoid biosynthesis and citrate cycle, indicating distinct metabolic adaptations that contribute to its unique flavor characteristics.

### 3.6 WGCNA of transcriptomic and metabolomic data

**Figure 6:**
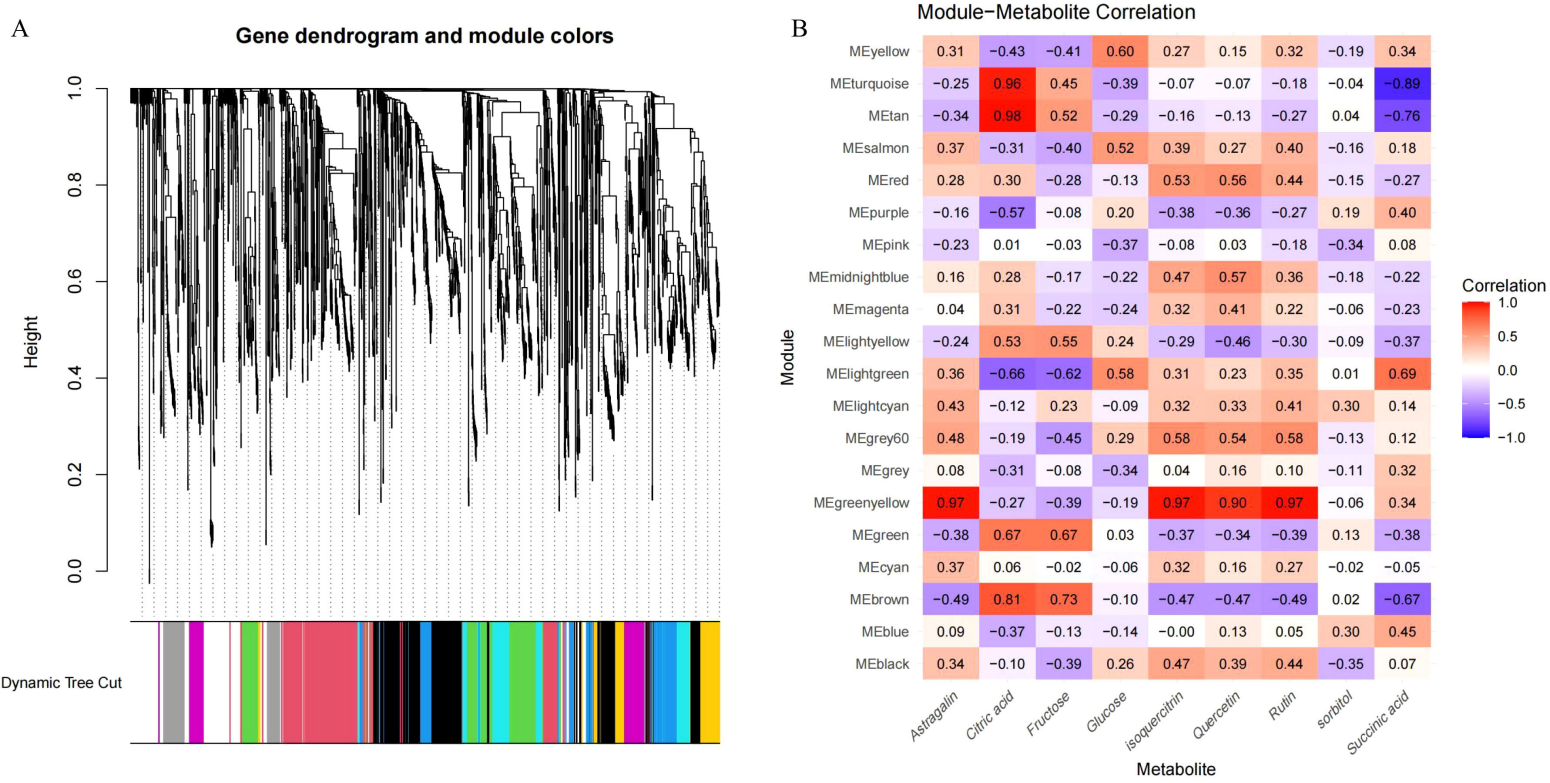
WGCNA of metabolomic data during the maturation process of Mei fruit. **(A)** Metabolite dendrogram and module colors; **(B)** module-metabolite correlation heatmap.

From the metabolomic WGCNA, we identified two modules, MEturquoise and MEtan, comprising 35 metabolites related to citric acid. Additionally, the module MEgreenyellow was associated with flavonoids, including astragalin, quercetin, and rutin (**Figure 6**). In the transcriptomic WGCNA, modules MEbisque4 and MEcoral1 were related to citrate acid metabolism, whereas the modules MEturquoise and MEthistle2 were linked to flavonoid metabolism (**Supplementary Figure S3**). Leveraging these data, we extracted relevant metabolites and genes from the identified modules. Using a correlation analysis, which included previously identified DEGs and DAMs, we explored potential regulatory pathways for acid and flavonoid metabolism during enlargement and green mature stages of Mei fruit.

### 3.7 Association analysis of the transcriptome and metabolome

**Figure 7:**
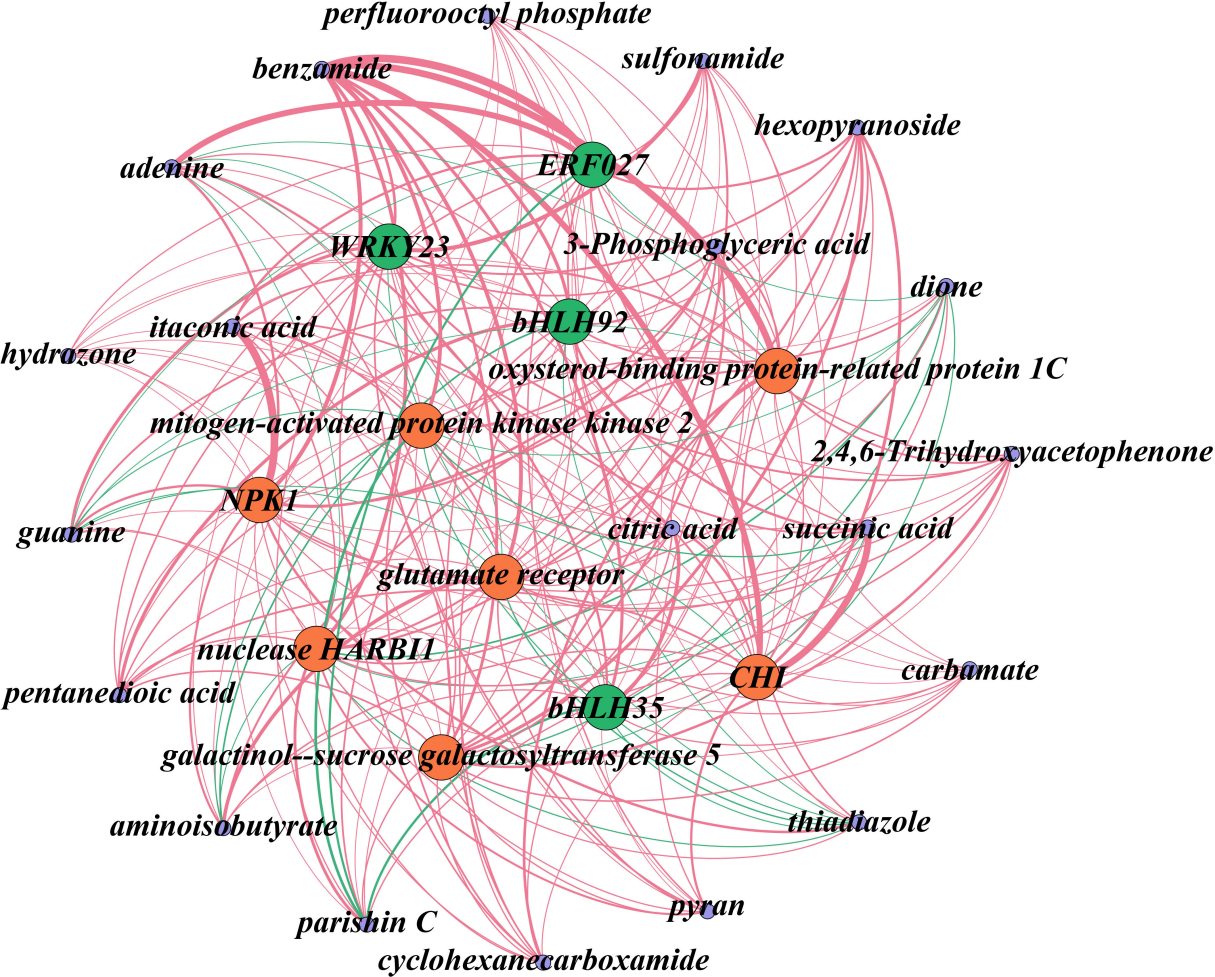
Network analysis of DEGs and DAMs associated with acid metabolism during the maturation of Mei fruit. In this network, transcription factors are denoted in green, functional genes in red, and metabolites in purple. Positive regulatory relationships are illustrated with red edges, while negative ones are marked in green. The thickness of the lines indicates the strength of the correlation, with thicker lines representing stronger correlations. *NPK1*, *mitogen-activated protein kinase kinase kinase NPK1-like*; *CHI*, *chalcone isomerase*.

The analysis highlighted several key metabolites, including citric acid, succinic acid, and itaconic acid, as potential primary contributors to the acidic taste of Mei fruit. These metabolites appeared to be regulated by genes such as galactinol-sucrose galactosyltransferase 5 (*GGT*), oxysterol-binding protein-related protein 1C (*ORP1*), *NPK1*, and *CHI*. Furthermore, transcription factors like *WRKY23*, *ERF027*, *bHLH92*, and *bHLH35* were identified as potential regulators within these networks (**Figure 7**). This network elucidation not only underscored the complexity of acid metabolism regulation in Mei fruit maturation but also pinpointed specific metabolites and genes that could be crucial for the development of the fruit’s acidic taste. The identified transcription factors represent intriguing targets for further functional studies aimed at understanding their roles in the modulation of flavor-related metabolic pathways.

### 3.8 Candidate genes linked to organic acid and flavonoid metabolisms

**Figure 8:**
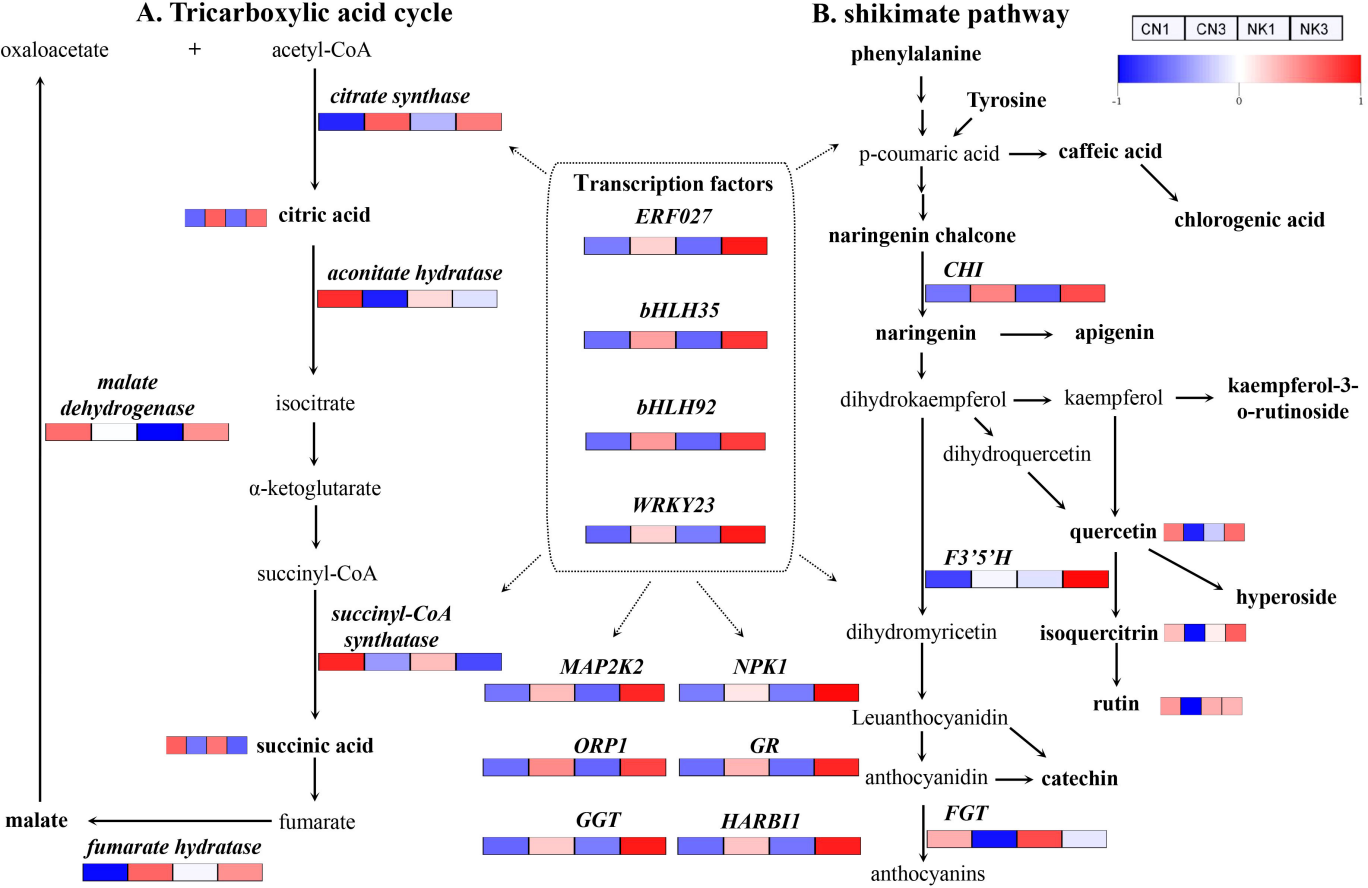
The tricarboxylic acid cycle **(A)** and shikimate pathway **(B)** in the two Mei fruit. FGT, flavonol-3-O-glucosidetransferase; F3’5’H, flavonoid 3’5’-hydroxylase; ORP1, oxysterol-binding protein-related protein 1C; GR, glutamate receptor; GGT, galactinol-sucrose-galactosyltransferase 5; HARBI1, nuclease HARBI1; CHI, chalcone isomerase; NPK1, mitogen-activated protein kinase kinase kinase NPK1-like; MAP2K2, mitogen-activated protein kinase kinase 2. The heatmap colors indicate the degree of regulation, with red representing upregulation, blue representing downregulation.

The DEG identification, correlation analysis, and the common KEGG pathway analyses of the DEGs and DAMs related to organic acid and flavonoid metabolisms identified candidate genes linked to the metabolism of organic acid and flavonoids (**Figure 8**; **Supplementary Table S3**). A heatmap containing each gene and metabolite was constructed to show their expression changes across different samples. For clarity, the heatmap values were horizontally standardized using the z-score method. Among them, DEGs, such as *ERF027*, *bHLH35*, *bHLH92*, *WRKY23*, *citrate synthase*, *fumarate hydratase*, *CHI*, and *F3’5’H*, and DAMs, such as citric acid, showed increasing trends in both CN and NK fruit from the enlargement stage to the green mature stage. In contrast, some DEGs, such as *aconitate hydratase*, *succinyl*-*CoA synthatase*, and *FGT*, and some DAMs, such as succinic acid and astragalin, decreased during CN and NK fruit ripening (**Figure 8**).

### 3.9 Analyses of organic acids, sugars, amino acids, and flavonoids

**Figure 9:**
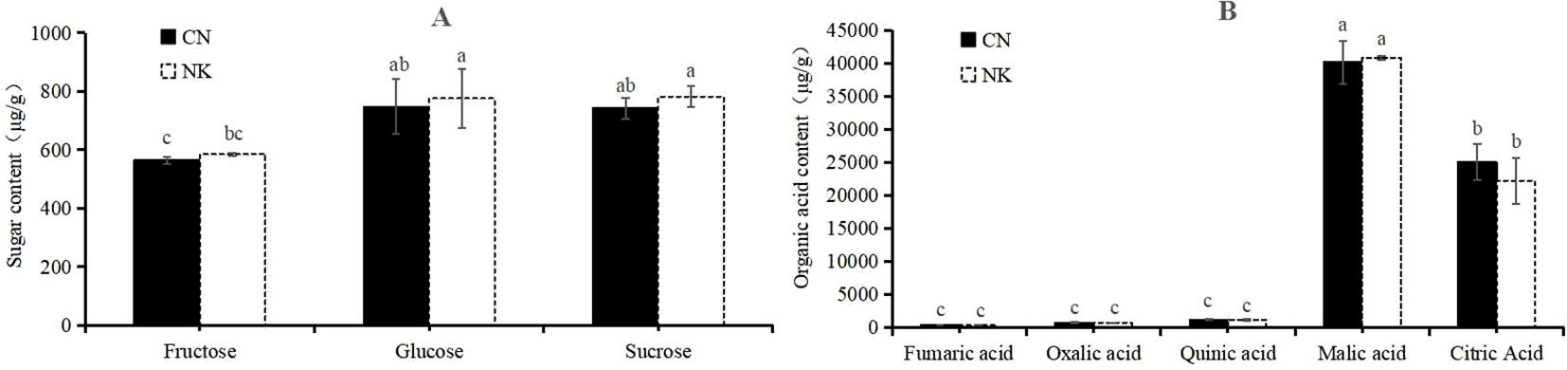
Sugars **(A)** and organic acids **(B)** contents in ‘Changlong 17’ (CN) and ‘Nankao’(NK) fruit at the green maturity stage. Short bars on the column chart indicate standard errors, and different letters indicate significant differences (*p*<0.05), as determined by Turky’s test.

Three sugars, fructose, glucose, and sucrose, and five organic acids, malic acid, citric acid, quinic acid, oxalic acid, and fumaric acid, were detected in the two Mei fruit at the green mature stage. Other sugars and organic acids were not detected due to their low contents. There were no significant differences between CN3 and NK3 in the detected sugars and acids, but the sucrose and glucose contents of the two Mei fruit were significantly higher than the fructose content. Among the organic acids, malic acid was the highest, followed by citric acid. The fumaric acid contents of CN3 and NK3 were relatively low, at 6.455 μg/g and 2.694 μg/g, respectively (**Figure 9**). In total, 20 amino acids were detected in the two Mei fruit at the green mature stage. Among them, the contents of isoleucine, valine, and threonine in NK3 were significantly higher than those in CN3, whereas the other 17 amino acids showed no significant differences between the two Mei fruit. The asparagine content was the highest, followed by aspartic acid, and the cysteine content was the lowest (**Supplementary Table S4**). A total of 41 flavonoids were detected in Mei fruits at the green mature stage, but only seven flavonoids were significantly different between CN3 and NK3. For example, the ginkgetin and apigenin contents in CN3 were significantly higher than in NK3, whereas the quercetin, hyperoside, rhoifolin, kaempferol-3-o-rutinoside, and rutin contents in NK3 were significantly higher than the corresponding values in CN3 (**Table 1**).

**Table 1:** Flavonoids contents (ng/g) of ‘Changlong 17’ (CN) and ‘Nankao’(NK) fruit at the green maturity stage.

Flavonoids	CN	NK
Psoralen	2.982 ± 0.813	2.086 ± 0.273
Hydroxychalcone	10.730 ± 0.903	9.323 ± 0.690
Isobavachalcone	2.663 ± 1.650	0.232 ± 0.269
Bavachin	–	4.320 ± 0.756
Tricin	1.665 ± 0.134	0.947 ± 0.551
Nobiletin	1.250 ± 0.353	0.959 ± 0.045
Mangiferin	4.410 ± 0.151	4.471 ± 0.225
Isomangiferin	4.687 ± 1.352	3.315 ± 0.830
Baicalin	22.261 ± 3.615	18.207 ± 1.120
Ginkgetin	4.565 ± 0.177^a^	1.467 ± 1.139^b^
Narirutin	1.615 ± 0.417	2.398 ± 1.587
Protocatechualdehyde	90.983 ± 30.792	109.733 ± 32.112
Protocatechuic acid	3.5673 ± 3.314	3.026 ± 2.946
Umbelliferone	4.118 ± 1.367	4.649 ± 2.133
Gallic acid	13.130 ± 4.475	11.817 ± 4.573
Caffeic acid	70.160 ± 20.156	76.598 ± 12.604
Apigenin	1.115 ± 0.431^a^	0.210 ± 0.101^b^
Galangin	3.640 ± 2.319	1.242 ± 0.922
Emodin	0.270 ± 0.014	–
Naringenin	4.977 ± 1.082	4.898 ± 1.884
Rhein	5.895 ± 2.064	2.842 ± 3.566
Kaempferol	74.302 ± 34.580	142.390 ± 3.389
Sakuranetin	5.497 ± 0.329	–
Epicatechin	34,680.157 ± 7,633.965	33,790.665 ± 12,629.656
Catechin	12,482.642 ± 911.200	11,072.801 ± 3,814.388
Farrerol	2.377 ± 0.422	–
Hispidulin	2.630 ± 1.053	1.450 ± 0.198
Hesperetin	–	3.833 ± 0.450
Quercetin	594.556 ± 102.236^b^	1,342.298 ± 43.869^a^
Aurantio-obtusin	1.120 ± 0.410	0.953 ± 0.508
Chlorogenic acid	12,099.472 ± 4148.612	12,297.927 ± 332.805
Vitexin	1.988 ± 0.563	1.557 ± 0.216
Isoorientin	1.663 ± 0.670	–
Ursolic Acid	16,548.668 ± 3,525.525	18,019.543 ± 4,370.610
Hyperoside	8,357.801 ± 2,155.211^b^	18,097.426 ± 584.565^a^
Rhoifolin	4.885 ± 0.074^b^	6.213 ± 0.216^a^
Naringin	4.815 ± 0.217	4.439 ± 0.278
Kaempferol-3-0-rutinoside	277.854 ± 62.172^b^	650.681 ± 59.047^a^
Tiliroside	2.300 ± 0.395	1.272 ± 0.990
Rutin	12,165.905 ± 1,817.826^b^	29,282.860 ± 1,780.349^a^
Isorhamnetin 3-O-neohesperidoside	85.090 ± 27.171	122.547 ± 29.988

The “–” in the table indicates that the content is very low and has not been detected by the instrument. Data are mean ± standard deviation, different letters in the same row indicate significant difference (*p* < 0.05), and no letter indicates no significant difference (*p* > 0.05), as determined by t-test.

### 3.10 Validation of candidate DEGs by qPCR

**Figure 10:**
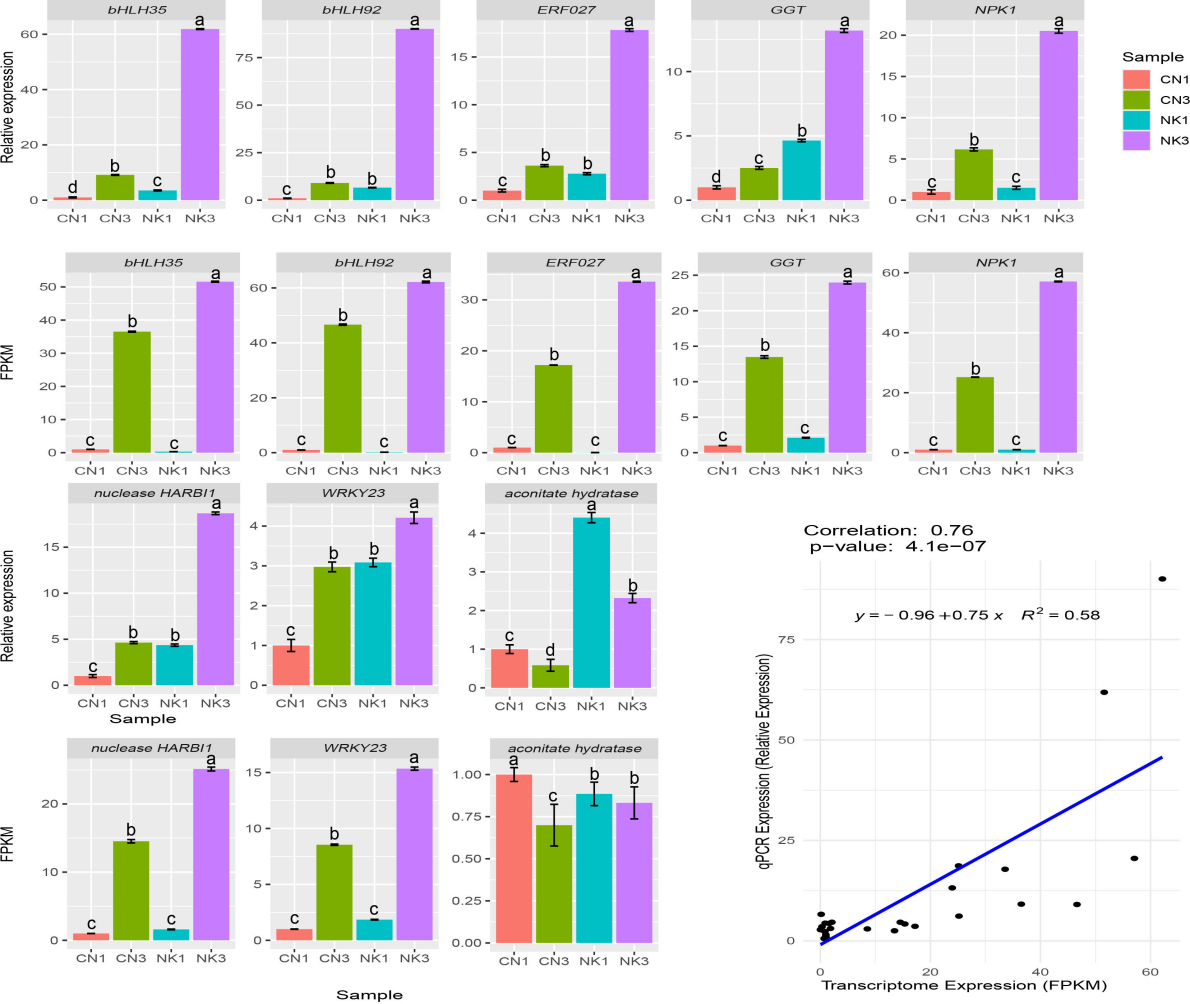
qPCR and RNA-Seq data analysis of eight DEGs in fruit of ‘Changlong 17’ (CN) and ‘Nankao’ (NK). Error bars were calculated based on three replicates. *GGT*, *galactinol-sucrose-galactosyltransferase 5*; *NPK1*, *mitogen-activated protein kinase kinase kinase NPK1-like*.

To validate the relative expression patterns of the unigenes, we selected eight key DEGs related to organic acid and flavonoid metabolism and performed a qPCR expression analysis between the two Mei fruit at the two developmental stages. The expression patterns of all eight key DEGs from the qPCR were similar to the RNA-Seq, confirming the reliability of the RNA-Seq data and subsequent analyses (**Figure 10**).

## 4 Discussion

The metabolism of organic acids is essential for fruit development, and their accumulation levels in fruit are central to fruit taste and quality. Thus, it is important to reveal the mechanisms involved in the organic acid metabolism of Mei fruit. In this study, significant gene expression differences were observed between the enlargement and green mature stages of CN and NK Mei fruit, with 861 DEGs identified for the CN Mei fruit (643 upregulated and 218 downregulated) and 457 for the NK Mei fruit (424 upregulated and 33 downregulated). This indicates a dynamic change in gene expression related to maturation, suggesting a complex regulatory network for Mei fruit flavor development. A PCA based on transcriptomic sequencing data further highlighted distinct separations between the cultivars and their developmental stages, with the emphasis revealing more significant varietal differences than maturation stage differences. GO annotation and KEGG pathway enrichment analyses of shared DEGs between CN and NK Mei fruit indicated significant enrichments in key functional categories associated with maturation and flavor development processes. Comparatively, existing literature on other fruits, like jujube, pear, hawthorn, and apricot, presents a varied landscape of differential gene expressions and metabolic pathways involved in maturation. For instance, a jujube study identified 16,245 DEGs enriched in pathways involved in glucosyltransferase activity and lipid binding (Lu et al., 2022), whereas a pear study emphasized terpenoid synthesis pathways (Li et al., 2023). A hawthorn study highlighted *CS* as a key gene (Wang et al., 2023), whereas an apricot study identified 1,237 DEGs, such as *sucrose synthase*, *sucrose phosphate synthase*, *invertase*, *NADP-dependent malic enzyme*, and *enolase*, that have significant impacts on sugar and organic acid metabolism. Moreover, genes like *carotenoid cleavage dioxygenase* and fatty acid desaturase have roles in synthesizing volatiles and lactones in apricot (Zhang et al., 2019), illustrating the intricate interactions within biochemical processes producing unique flavors during fruit maturation. Our findings align with these studies in highlighting the importance of a broad spectrum of metabolic pathways and genes involved in fruit maturation and flavor development. However, our study uniquely identified a specific set of metabolites and genes, such as citric acid, succinic acid, quercetin, and rutin, and key functional genes, which are directly associated with the acid and flavonoid profiles of Mei fruit. The emphasis on acid metabolism-related pathways and their regulatory genes provides new insights into the biochemical foundations of flavor development in Mei fruit, and this differs from the focus areas of previous studies on other fruit.

In our study, by analyzing transcriptomic and metabolomic data across the two Mei fruit, we identified DEGs. We then conducted a WGCNA to pinpoint genes and metabolites associated with organic acid and flavonoid metabolism. We hypothesized that metabolites and genes related to citric acid, which showed differential expression during fruit maturation, play crucial roles in the acid characteristic-related regulation of Mei fruit. Our key findings indicate that malic acid, citric acid, succinic acid, quinic acid, oxalic acid, and fumaric acid are likely the primary metabolites associated with the sour taste of Mei fruit. In the CN and NK Mei fruit, malic acid and citric acid were the main organic acids in the green mature stage, with the content of malic acid being the highest. The content of malic acid was higher than that of citric acid in the early stage of Mei fruit ripening, whereas the content of citric acid was higher than that of malic acid in the late stage of Mei fruit ripening (Liu et al., 2017). These results are consistent with those shown in **Figure 8**. Additionally, there was no significant difference in the sugar content between CN3 and NK3, but the sucrose and glucose contents of the two Mei fruit were significantly higher than the fructose contents (**Figures 1**, **9**). Among the six essential amino acids, the threonine content was the highest, especially in NK3, whereas the methionine content was the lowest (**Supplementary Table S4**). However, Lin et al. (2014) reported that as Mei fruit matures, amino acids abundantly accumulate. This difference is closely related to the different maturation levels of Mei fruit. In this study, from the enlargement to the green mature stages of Mei fruit development, the citric acid content significantly increased, whereas the succinic acid content significantly decreased, which was consistent with the expression of some genes related to the tricarboxylic acid cycle. For example, the key enzyme for synthesizing citric acid, encoded by the *CS* gene, was significantly upregulated, whereas the enzyme for degrading citric acid, encoded by the *aconitate hydratase* gene, and the key enzyme for synthesizing succinic acid, encoded by the *succinyl-CoA synthatase* gene, were significantly downregulated (**Figure 8**). Similarly, in Mei fruit, seven phenolic acids (neochlorogenic, isoferulic, p-coumaric, chlorogenic, cryptochlorogenic, p-hydroxybenzoic, and ferulic) and three flavonoids (epicatechin, epicatechin B1, and rutin) have been identified. Among them, monomers, such as anthocyanin B1, rutin, p-coumaric acid, and ferulic acid, may be the main effective substances behind the antioxidative capacity (Yang et al., 2022b). The increase in the citric acid content of lemon fruit is related to an increase in citrate synthase activity and a decrease in degrading enzyme, such as aconitate hydratase activity (Zhang and Chen, 2007). In addition, the gene encoding the enzyme fumarate hydratase, which catalyzes fumarate to malate, was upregulated with ripening in both Mei fruit; however, the enzyme malate dehydrogenase, which catalyzes the conversion of malate to oxaloacetate, and the gene *malate dehydrogenase* were downregulated in CN but upregulated in NK as fruit ripened (**Figure 8**). Similarly, Gao et al. (2022) reported that 19 genes, *including aconitase*, *CS*, *malate dehydrogenase*, and *glutamate decarboxylase*, likely play important roles in citric acid accumulation during pineapple fruit development.

In the present study, functional genes, such as *mitogen-activated protein kinase kinase 2*, *NPK1*, *ORP1*, *GGT*, *glutamate receptor*, and *nuclease HARBI1*, were identified as being significant in regulating acid and flavonoid metabolisms, with transcription factors *ERF027*, *bHLH35*, *bHLH92*, and *WRKY23*, playing pivotal roles in these processes. Unlike previous studies on apricots (Zhang et al., 2019) where genes involved in sucrose synthase and carotenoid metabolisms were more prominent, and similar transcription factors such as *MYBs* and *bHLHs* have been implicated in flavonoid biosynthesis in strawberries (Liu et al., 2023). ERF participates in resistance to diseases, responses to abiotic stresses, such as drought, and molecular responses to ethylene; WRKY is widely involved in regulating plant defense responses and growth and development processes; and bHLH participates in the signal transduction of phytochrome and can bind with MYB to participate in anthocyanin synthesis. Similarly, *MYB110*, *MYB108*, and *MYB44* were particularly highly expressed during *Vaccinium bracteatum* fruit maturation (Yang et al., 2022a). *MYBA* may regulate the differences between blue and black blueberries by changing the expression levels of some structural genes in the anthocyanin biosynthesis pathway (Chu et al., 2024). MYBs and bHLHs may be involved in the formation of fruit acidity and anthocyanins of interspecific grafted blueberry (Zhu et al., 2024) and grapevine (Zhong et al., 2022). Zhang et al (2019) identified an ERF interaction factor, MYB98, that may regulate flavor formation in apricot. In the shikimate pathway, the accumulation of the metabolites quercetin, isoquercitrin, and rutin in CN fruit obviously decreased with fruit ripening. This was in agreement with the changes in the expressions of related genes, such as *FGT*, which decreased with fruit ripening. However, *CHI* in both Mei fruit and *F3’5’H* in NK fruit were drastically upregulated with fruit ripening. This was consistent with the significant increase in the anthocyanin content of NK fruit and the obvious accumulations of quercetin and isoquercitrin in NK fruit (**Figures 1**, **8**). This was also consistent with the results shown in **Table 1**, such as the significantly higher contents of quercetin, rutin, hyperoside, vernicin, and kaempferol-3-o-rutinoside in NK3 compared with the corresponding values in CN3. Rutin, also known as vitamin P, has powerful antioxidant and anti-inflammatory effects. Quercetin has antioxidant, anti-inflammatory, antiviral, anti-tumor, immune regulation, and other biological activities (Zou et al., 2023 **;** Zhang and Chen, 2007). In general, epicatechin, catechin, ursolic acid, chlorogenic acid, hyperoside, rutin, and quercetin were the most abundant flavonoids in the two Mei fruit (**Table 1**). The results were similar to those for polyphenols in Mei fruit detected by Yang et al. (2023). Catechin and epicatechin have antioxidant, immunomodulatory, and antitumor functions. Chlorogenic acid is an important bioactive substance that has antibacterial, antiviral, antitumor, antihypertensive, hypolipidemic, and other effects. Hyperoside, also known as quercetin-3-o-β- D-galactopyranoside, has obvious anti-inflammatory and antitussive effects, and it may also be beneficial to the prevention of diabetic cataracts (Zhang and Chen, 2007). Thus, Mei fruit containing these flavonoids have high health values. Similarly, the ion abundances of kaempferol, chlorogenic acid, naringenin, rutin, and isoquercitrin decreases in *P. mume* with flower development, whereas the ion abundance of cinnamic acid increases (Wu et al., 2024). The walnut–bacterial blight interaction induced the expression of polyphenol oxidase gene *JrPPO-1* and pathogenesis-related gene *P14a* and the activity of polyphenol oxidase (Khodadadi et al., 2016, Khodadadi et al., 2020). Some structural and regulatory genes, such as *F3’H* and *F3’5’H*, which are involved in the anthocyanin biosynthetic pathway, are upregulated during the late stage of blueberries development (Gao et al., 2022). Several flavonoid biosynthesis-related genes such as *chalcone synthase* were significantly upregulated, suggesting their essential functions in the accumulation of flavonoids in maturing acerola cherry fruit (Xu et al., 2022). The overexpression of the *PmUFGT3* allele from red-skinned Japanese apricot results in greater anthocyanin accumulations in fruit skin (Ni et al., 2022).

Our study’s comprehensive WGCNA of metabolomic and transcriptomic data during Mei fruit maturation illuminated the intricate regulation of sugar–acid metabolism and identified key metabolic traits associated with acid and flavonoid pathways. This suggests variations in flavor development mechanisms in different fruits. Compared with existing literature, our findings revealed both similarities and distinctions in the regulatory mechanisms of fruit flavor. For instance, in jujube, key transcription factors related to sugar–acid metabolism include *HAP3*, *TCP14*, and *MYB78* (Lu et al., 2022), whereas in apple, *NAC1* emerges as a primary flavor-regulating transcription factor (Wang et al., 2024), and *MYB94* participates in the regulation of ester biosynthesis (Li et al., 2023b). Pear studies have highlighted the mevalonate and 2-c-methyl-d-erythritol 4-phosphate pathways as critical to volatile organic compound-related metabolic routes (Li et al., 2023a). In apricot, 16 transcription factors may play roles in flavor development (Zhang et al., 2019). *MYB*s and *bHLH*s may mediate the changes in flavonoid contents in the embryogenic cultures of longan (Lai et al., 2023). These results underscore the diversity of genetic and metabolic pathways contributing to fruit flavor across different species. Our work adds to this body of knowledge by pinpointing specific metabolites (e.g., citric acid, succinic acid) and modules (MEturquoise and MEtan) that play significant roles in the sour taste characteristic of Mei fruit. This specificity in the context of Mei fruit maturation offers new insights, particularly in the regulation of acid metabolism, distinguishing our findings from those in other fruit in which flavor development may focus on different metabolic pathways or regulatory genes. The unique significance of our study lies in its detailed analysis of the regulatory network underlying sugar–acid metabolism in Mei fruit, which provides a focus on the metabolic and genetic foundations of acid taste formation.

Our integrated network analysis, developed from comprehensive metabolomic and transcriptomic data, has led us to postulate a novel regulatory mechanism for acid control in Mei fruit. This hypothesis is grounded in the observed significant associations between specific metabolites, such as citric acid and succinic acid, and key regulatory genes, including *GGT* and *glutamate receptor*, which are further supported by transcription factors like *WRKY23* and *ERF027*. Our network suggests potential cross-talk between these pathways and the acid metabolism in Mei fruit. This interaction is hypothesized to contribute to the accumulation of acidic compounds, which are pivotal for the distinctive sour flavor of the fruit, and genes such as *CHI*, which catalyzes the production of naringenin from chalcone in the metabolic process of synthesizing anthocyanins through the shikimate pathway. This central hub in our network may serve dual functions in both the regulation of flavonoid pathways and modulation of acid-related metabolites. While these connections have not been experimentally validated and thus remain speculative, the hypothesized mechanism presents a new perspective on the complex biosynthetic networks that govern flavor and aroma.

## Data Availability

Sequencing data used in this study are available in the NCBI Sequence Read Archive database (accession numbers: PRJNA1096442).

## Supplementary material

Supplementary Figure 1 Images of ‘Changnong 17’ (CN) and ‘Nanko’ (NK) fruit at the enlargement stage and green mature stage

Supplementary Figure 2 The flowchart of the major analyses

Supplementary Figure 3 WGCNA of transcriptomic data during the maturation process of Mei fruit. **(A)** Gene dendrogram and module colors; **(B)** Module-gene correlation heatmap. *AH*, *aconitate hydratase*; *CS*, *citrate synthase*; *F3’5’H*, *flavonoid 3’5’-hydroxylase*; *FGT*, *flavonol-3-O-glucosidetransferase*; *FH*, *fumarate hydratase*; *MDH*, *malate dehydrogenase*; *SS*, *succinyl-CoA synthetase*.
