# Supplementary figures and images for: The key metabolic genes and networks regulating the fruit acidity and flavonoid of Prunus mume revealed via transcriptomic and metabolomic analyses
Source: Front Plant Sci. 2025 Jan 31;16:1544500. doi: 10.3389/fpls.2025.1544500 (PMC11825340; doi:10.3389/fpls.2025.1544500)

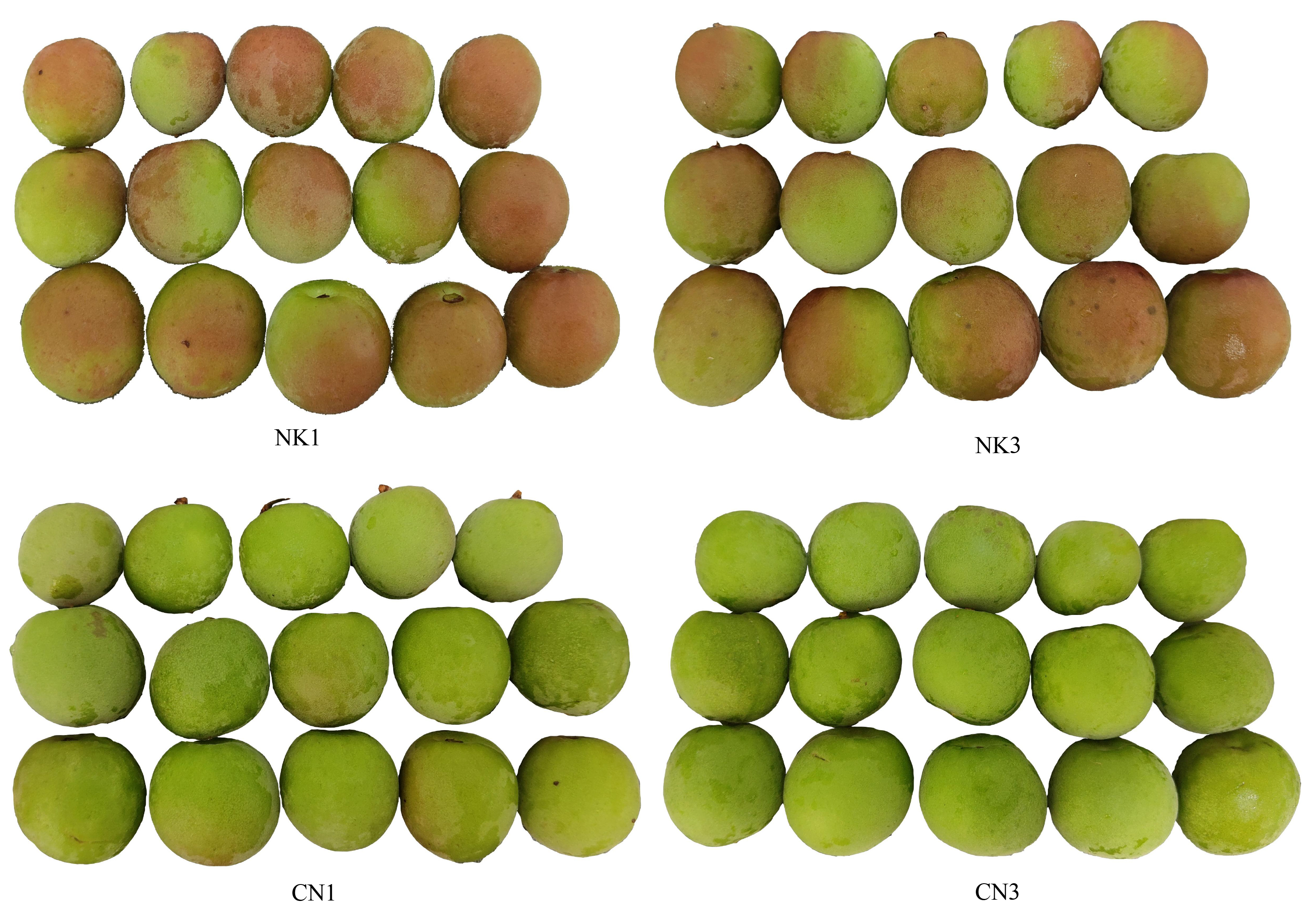

Supplement: Supplementary Figure 1 — Images of ‘Changnong 17’ (CN) and ‘Nanko’ (NK) fruit at the enlargement stage and green mature stage [file Image1.jpeg]

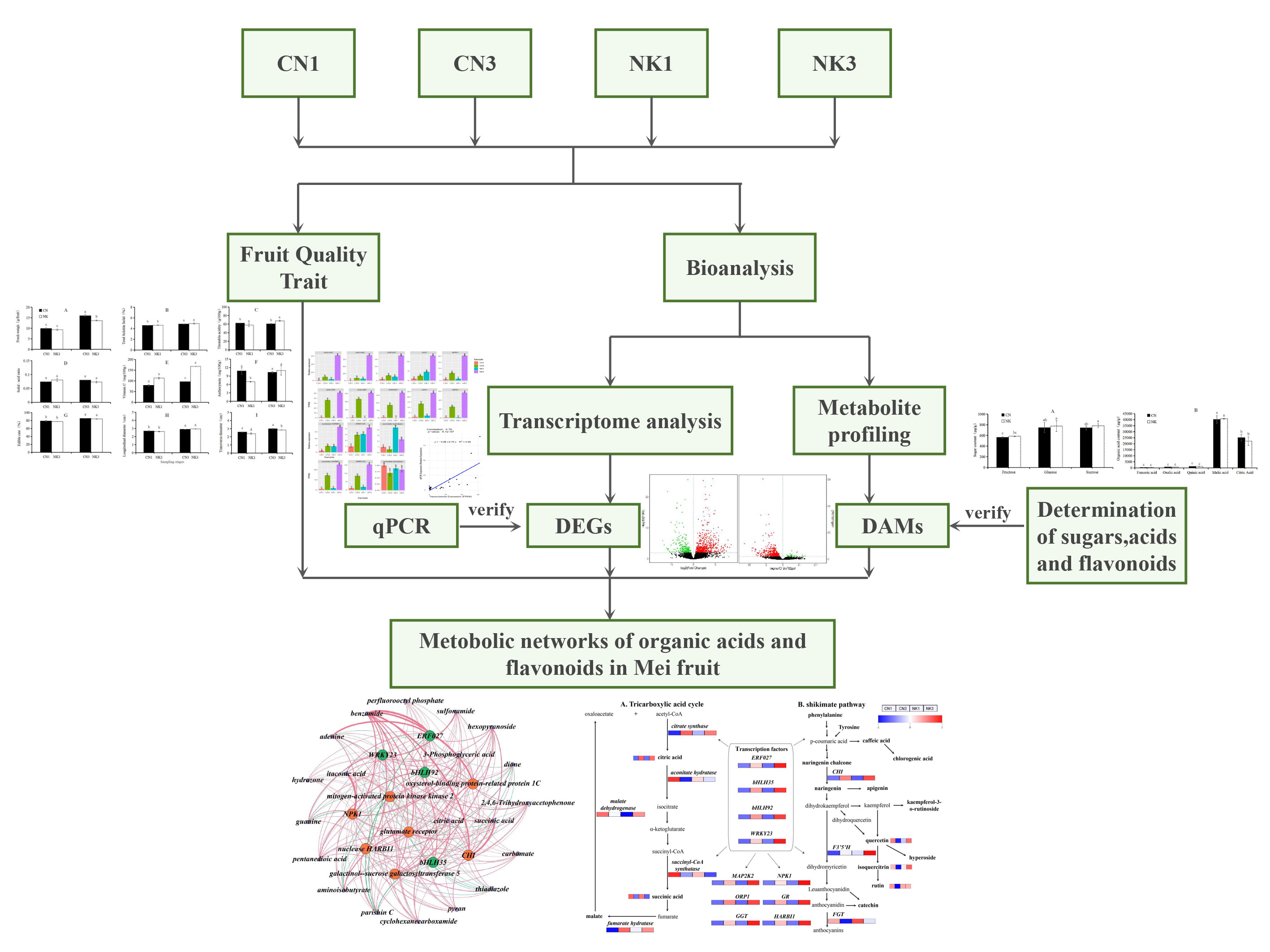

Supplement: Supplementary Figure 2 — The flowchart of the major analyses [file Image2.jpeg]

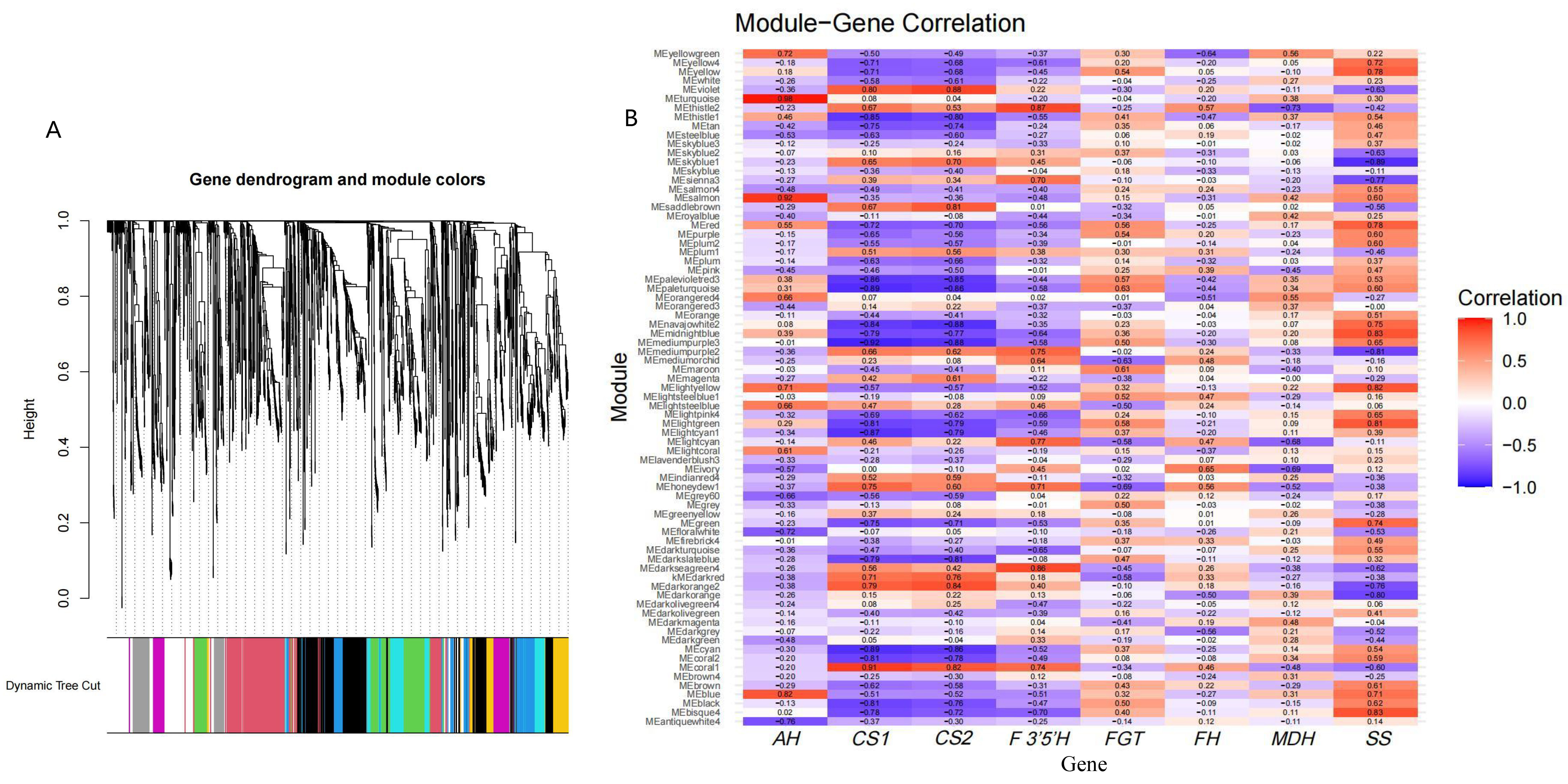

Supplement: Supplementary Figure 3 — WGCNA of transcriptomic data during the maturation process of Mei fruit. (A) Gene dendrogram and module colors; (B) Module-gene correlation heatmap. AH, aconitate hydratase; CS, citrate synthase; F3’5’H, flavonoid 3’5’-hydroxylase; FGT, flavonol-3-O-glucosidetransferase; FH, fumarate hydratase; MDH, malate dehydrogenase; SS, succinyl-CoA synthetase. [file Image3.jpeg]
