# Supplementary material for: The key metabolic genes and networks regulating the fruit acidity and flavonoid of Prunus mume revealed via transcriptomic and metabolomic analyses
Source: Front Plant Sci. 2025 Jan 31;16:1544500. doi: 10.3389/fpls.2025.1544500 (PMC11825340; doi:10.3389/fpls.2025.1544500)
Supplement: Supplementary file 4 [file Table1.doc]

**Table S1 qPCR genes and primers used for validation of RNA-Seq data**

| Gene ID | Gene name | Forward primer(5’→3’) | Reverse primer(5’→3’) |
| --- | --- | --- | --- |
| 103320135 | *LOC103320135* | CAAACCCCGGACTCAAAAAC | ATCTTCAGAGCGGACGAATC |
| 103323643 | *bHLH35* | GAGCAGTGGTCCCCAAAATA | TAGCCACCACTGTCTTTTCC |
| 103324163 | *bHLH92* | ATCCTTGAAGCACTGGAAGG | GTCCTCTCCGGTTACGATTC |
| 103337405 | *ERF027* | CTTGACTTTCACCCTCCTCC | GTCAACCTCTAGTGCTCTCG |
| 103329815 | *galactinol-sucrose galactosyltransferase 5* (*GGT*) | AAACGGCCATGTCATTCTCT | GCTTTCGAGGATGACGATCT |
| 103326494 | *mitogen-activated protein kinase kinase kinase NPK1-like* (*NPK1*) | TACGATTCGAAGGCATGGAG | TTCAAACCCCTGCTCTTCTC |
| 103320616 | *nuclease HARBI1* | CCAACAATGGCTTGCTGAAA | GCAATACCACCTCCCTTTCA |
| 103323186 | *WRKY23* | TGAAGCTGCTGCCAATAAGA | CCTGGGATAAGGGCTGTTTT |
| 103340381 | *aconitate hydratase 1* | AATGCTACTGGGTGTGAAGG | GTAACATCCTGTCCGGGTTT |

**Table S2** Summary of the transcriptome data of ‘Changnong 17’ (CN) and ‘Nanko’ (NK) fruit

| Sample | Raw Reads (M) | Clean Reads (M) | Alignment to a Reference Sequence (%) | Q30 Rate (%) |
| --- | --- | --- | --- | --- |
| CN1-1 | 43.69 | 42.35 | 96.55 | 92.06 |
| CN1-2 | 43.69 | 42.11 | 96.52 | 92.23 |
| CN1-3 | 43.69 | 42.44 | 96.74 | 91.66 |
| CN3-1 | 43.69 | 42.38 | 96.56 | 92.03 |
| CN3-2 | 43.69 | 42.52 | 96.70 | 91.31 |
| CN3-3 | 43.69 | 42.36 | 96.71 | 92.39 |
| NK1-1 | 43.69 | 42.67 | 96.44 | 93.08 |
| NK1-2 | 45.44 | 43.54 | 96.37 | 92.57 |
| NK1-3 | 47.19 | 45.76 | 96.85 | 93.56 |
| NK3-1 | 43.69 | 42.14 | 96.30 | 90.34 |
| NK3-2 | 43.69 | 42.52 | 96.59 | 92.17 |
| NK3-3 | 43.69 | 42.47 | 96.81 | 91.57 |

**Table S3** The key DEGs and DAMs involved in key pathways

| Category | Gene/Metabolite ID | Gene/Metabolite | Pathway |
| --- | --- | --- | --- |
| DEGs | 103340381 | aconitate hydratase | Citrate cycle (TCA cycle) |
| DEGs | 103330589 | citrate synthase1 | Citrate cycle (TCA cycle) |
| DEGs | 103343519 | citrate synthase2 | Citrate cycle (TCA cycle) |
| DEGs | 103344194 | flavonoid 3',5'-hydroxylase | Flavone and flavonol biosynthesis |
| DEGs | 103343492 | flavonol-3-O-glucoside | Flavone and flavonol biosynthesis |
| DEGs | 103333998 | fumarate hydratase | Citrate cycle (TCA cycle) |
| DEGs | 103331480 | malate dehydrogenase | Carbon fixation in photosynthetic organisms |
| DEGs | 103334437 | succinyl-CoA synthetase | Citrate cycle (TCA cycle) |
| DAMs | HMDB0037429 | astragalin | Flavonoid biosynthesis |
| DAMs | HMDB0000094 | citric acid | Citrate cycle (TCA cycle) |
| DAMs | HMDB0001076 | fructose | Glycolysis / Fructose and mannose metabolism |
| DAMs | HMDB0001401 | glucose | Starch and sucrose metabolism |
| DAMs | HMDB0037362 | isoquercitrin | Flavonoid biosynthesis |
| DAMs | 7.458_507.11340 | quercetin | Flavonoid biosynthesis |
| DAMs | HMDB0003249 | rutin | Flavonoid biosynthesis |
| DAMs | Reference7439 | sorbitol | Sugar alcohol metabolism |
| DAMs | HMDB0000254 | succinic acid | Citrate cycle (TCA cycle) |

**Table S4** Amino acids contents (μg/g) in ‘Changlong 17’ (CN) and ‘Nankao’(NK) fruit at the green maturity stage

Note: *Essential amino acids; Data are mean ± standard deviation, different letters in the same row indicate significant difference (*p* < 0.05), and no letter indicates no significant difference (*p* > 0.05).

|  | CN | NK |
| --- | --- | --- |
| Glutamine | 2.050±0.366 | 1.938±0.357 |
| Asparagine | 892.437±143.797 | 724.681±54.438 |
| Tryptophan* | 1.294±0.450 | 3.009±1.276 |
| Cysteine | 0.261±0.056 | 0.472±0.157 |
| Leucine | 61.051±5.204 | 81.225±13.769 |
| Isoleucine | 11.391±0.699**^b^** | 24.755±7.789**^a^** |
| Glycine | 24.719±1.570 | 27.302±8.805 |
| Alanine | 111.526±21.125 | 119.498±18.299 |
| Serine | 30.093±1.485 | 35.518±13.325 |
| Proline | 32.527±2.856 | 32.003±5.656 |
| Valine* | 12.786±0.912**^b^** | 24.595±6.051**^a^** |
| Threonine* | 138.978±10.188**^b^** | 191.558±30.659**^a^** |
| Aspartate | 339.282±34.277 | 363.495±24.571 |
| Lysine* | 3.672±0.799 | 3.322±0.423 |
| Glutamate | 135.170±28.686 | 188.517±38.657 |
| Methionine* | 0.750±0.068 | 0.947±0.140 |
| Histidine | 133.979±38.375 | 142.167±41.659 |
| Phenylalanine* | 28.734±3.747 | 35.875±6.633 |
| Arginine | 110.444±20.120 | 78.492±16.426 |
| Tyrosine | 22.947±3.475 | 35.941±15.428 |
